# Identification of Kernel Proteins Associated with the Resistance to *Fusarium* Head Blight in Winter Wheat (*Triticum aestivum* L.)

**DOI:** 10.1371/journal.pone.0110822

**Published:** 2014-10-23

**Authors:** Dawid Perlikowski, Halina Wiśniewska, Tomasz Góral, Michał Kwiatek, Maciej Majka, Arkadiusz Kosmala

**Affiliations:** 1 Institute of Plant Genetics of the Polish Academy of Sciences, Poznan, Poland; 2 Plant Breeding and Acclimatization Institute – National Research Institute, Radzikow, Blonie, Poland; The University of Melbourne, Australia

## Abstract

Numerous potential components involved in the resistance to *Fusarium* head blight (FHB) in cereals have been indicated, however, our knowledge regarding this process is still limited and further work is required. Two winter wheat (*Triticum aestivum* L.) lines differing in their levels of resistance to FHB were analyzed to identify the most crucial proteins associated with resistance in this species. The presented work involved analysis of protein abundance in the kernel bulks of more resistant and more susceptible wheat lines using two-dimensional gel electrophoresis and mass spectrometry identification of proteins, which were differentially accumulated between the analyzed lines, after inoculation with *F. culmorum* under field conditions. All the obtained two-dimensional patterns were demonstrated to be well-resolved protein maps of kernel proteomes. Although, 11 proteins were shown to have significantly different abundance between these two groups of plants, only two are likely to be crucial and have a potential role in resistance to FHB. Monomeric alpha-amylase and dimeric alpha-amylase inhibitors, both highly accumulated in the more resistant line, after inoculation and in the control conditions. *Fusarium* pathogens can use hydrolytic enzymes, including amylases to colonize kernels and acquire nitrogen and carbon from the endosperm and we suggest that the inhibition of pathogen amylase activity could be one of the most crucial mechanisms to prevent infection progress in the analyzed wheat line with a higher resistance. Alpha-amylase activity assays confirmed this suggestion as it revealed the highest level of enzyme activity, after *F. culmorum* infection, in the line more susceptible to FHB.

## Introduction

*Fusarium* species are widespread necrotrophic pathogens of small grain cereals, e.g. oat (*Avena sativa* L.), wheat (*Triticum aestivum* L.) and triticale (×*Triticosecale* Wittm.). Three of these species – *F. avenaceum* (Corda ex Fries) Sacc., *F. culmorum* (W.G. Smith) Sacc. and *F. graminearum* (Schwabe.) are considered to be the most important in central European countries [1]. Severity of *Fusarium* head blight (FHB) depends on several agronomic, climatic and genetic factors [2]–[4]. This disease can result in *Fusarium*-damaged kernels (FDK), which are smaller, compared to healthy-looking kernels (HLK), discoloured and shriveled [5]–[7]. Accumulation of *Fusarium* toxins such as deoxynivalenol (DON), nivalenol (NIV), zearalenone and many others in infected chaff, kernels and rachises is also often observed [8]–[10]. Contamination of the harvested grain with toxic fungal secondary metabolites (mycotoxins) may cause mycotoxicoses in humans and domestic animals [11], [12]. Observations of FHB occurrence revealed a high susceptibility of cultivars and breeding lines of spring wheat and oat to most *Fusarium* pathogens [13], [14]. Most of the published papers on triticale situate this species in terms of resistance between wheat and rye (*Secale cereale* L.). However, there are results available showing that susceptibility of triticale to FHB may be higher and even equal to wheat [15]–[17]. Under conditions of artificial inoculation with *F. culmorum* most winter wheat cultivars proved to be susceptible or highly susceptible to FHB, when compared to the known resistant winter wheat, e.g. ‘Arina’ or ‘SVP’ lines [18], [19]. Moreover, high yielding winter wheat cultivars that are best adapted to environmental conditions are often susceptible to FHB. The development of cultivars resistant to FHB plays a key role in disease control and the prevention of kernel contamination with mycotoxins [20], [21].

The resistance of wheat to FHB has a relatively complex nature. Five types of physiological resistance have been described [5]: type I or resistance to the initial infection, type II or resistance to spread within the spike, type III or resistance to kernel infection, type IV or tolerance to infection and type V or resistance to DON accumulation. However, the detailed defense mechanisms against FHB infection remain poorly characterized. An interaction between the pathogen and the host causes a defense response involving: hypersensitive reactions, deposition of cell wall reinforcing materials and synthesis of a wide range of antimicrobial compounds, such as pathogenesis-related (PR) proteins [22]. Gene expression studies revealed that the transcripts of defense response genes, coding peroxidase and PR-1-5, accumulated as early as six to 12 hours after inoculation of wheat spikes with *F. graminearum* [23]. Gottwald et al. [24] assumed that jasmonate and ethylene dependent defense and suppression of fungal virulence factors could provide major mechanisms of FHB resistance in wheat. In addition, proteomic studies have been carried out in *F. graminearum* infected wheat, barley (*Hordeum vulgare* L.) and their wild relatives [25]–[28]. Zhou et al. [29], [30] performed research on the interaction between *F. graminearum* and wheat to identify FHB infection response proteins by comparing protein profiles of *F. graminearum*-inoculated with mock-inoculated wheat spikelets of ‘Ning7840’, *Fhb1* resistance gene carrier. Gel-based proteomic analysis of the resistant cultivar revealed accumulation of plant proteins involved in oxidative stress, PR responses, and nitrogen metabolisms. The results showed up-regulation of proteins in the antioxidant and jasmonic acid-signaling pathway, PR responses and amino acid synthesis after three days of inoculation [29], [30].

Although, numerous potential components involved in the resistance to FHB have been indicated, our knowledge regarding this process in cereals is still limited and further work required. Here, we present comprehensive research on winter wheat, performed to recognize the crucial proteins associated with the resistance. Thus, the current work involved two main proteomic steps: (1) the analysis of protein abundance in the FDK of more resistant and more susceptible wheat lines using two-dimensional gel electrophoresis (2-DE) and (2) mass spectrometry (MS) identification of proteins which were differentially accumulated between the analyzed lines. It is hypothesized here that between the FDK derived from the lines with distinct levels of resistance, differentially accumulated proteins will be identified. Moreover, it is also suggested that the proteins highly accumulated in the more resistant line after *Fusarium* infection, crucial for the resistance, will have also higher abundance in this line in the control conditions, without infection. The procedure of proteome profiling was further followed by alpha-amylase activity assays performed on the FDK and on the kernels in the control conditions, both in the more resistant and more susceptible wheat lines to decipher involvement of this enzyme activity and its inhibition in the resistance of winter wheat to FHB.

## Materials and Methods

The study presented here does not require an ethics statement. The field plots described below, in Cerekwica and Radzikow, belong to the Institute of Plant Genetics of the Polish Academy of Sciences and to the Plant Breeding and Acclimatization Institute – National Research Institute, respectively. No special permissions were required to perform trials on these field plots and the trials did not involve endangered or protected species.

### Field experiments

The plant materials for the proteomic research were two lines of winter wheat (*Triticum aestivum* L.) – STH 0290, a line with a higher level of resistance to FHB (abbreviated here as ‘the resistant line’, RL), developed by Plant Breeding Strzelce Ltd., Co. (Poland) and AND 775/09, a line with a lower level of resistance (abbreviated here as ‘the susceptible line’, SL), developed by Danko Plant Breeding Ltd., Co. (Poland). The level of resistance of these two lines was estimated under the field conditions in 2013, in two locations: Cerekwica (western Poland; GPS coordinates: N 52.521012, E 16.692005) characterized by poor, sandy-clay soil and Radzikow (central Poland; GPS coordinates: N 52.211754, E 20.631954) with rich sandy-clay soil. The rainfalls and mean temperature during the experiments performed in Cerekwica and Radzikow, are presented in Table S1. The experiments in both locations were carried out according to the same design. The experimental field in each location consisted of four plots for each tested line. The seeds were sown in plots of 1 m^2^ size with the sowing rate 300 seeds (September 2012). The fungal material for inoculation was a mixture of three isolates of *F. culmorum* (W.G.Sacc.): KF 846 (DON chemotype) and KF 350 (NIV chemotype) derived from the collection of Institute of Plant Genetics, Polish Academy of Sciences (Poznan, Poland), and ZFR 112, producing zearalenone, derived from the collection of Plant Breeding and Acclimatization Institute – National Research Institute (Radzikow, Poland). In the case of each analyzed line, one plot, used as a control, was not treated with *Fusarium* isolates, and three others were inoculated by spraying flowering heads (stage of development: full flowering, 50% of anthers mature; stage 65 in commonly used BBCH scale) with the spore suspension at a rate of approximately 100 ml m^−2^ (June 2013). Conidia concentration was adjusted to 5×10^4 ^ml^−1^. During two days after inoculation micro-irrigation was applied to maintain moisture levels. Two weeks after inoculation, progress of the disease was visually evaluated. The presence of FHB (percentage of heads infected per plot) and percentage of head infection were determined. On the basis of the obtained results, FHB index (FHBi), associated with the resistance type I and II, as described by Mesterházy [5], was calculated, separately for each line (the RL and SL) and location (Cerekwica and Radzikow), according to the formula: FHBi (%) = (% of head infection×% of heads infected per plot)/100.

At the harvest (August 2013) 20 randomly selected heads from each plot (one control plot and three inoculated plots, in each location) were collected for the RL and the SL and threshed manually. Further, within a group of each 20 heads derived from three inoculated plots for each line mature kernels were visually scored and divided into two categories: HLK and FDK. In each category kernel weight [g] and number were recorded. Percentage of FDK (% FDK) was estimated as a percent of infected kernels per head, taking into account both kernel weight and number. Mean values and standard deviations of this parameter calculated on the base of data obtained from three inoculated plots were described in the paper, separately for each experimental location and each analyzed line. The percentage of FDK parameter is associated with the level of resistance type III, as described by Mesterházy [5]. Additionally, total kernel number and weight per head on the base of 20 randomly selected head from one control plot, separately for each analyzed line and location, were also calculated.

### Proteome profiling and identification of differentially accumulated proteins

**Figure 1:**
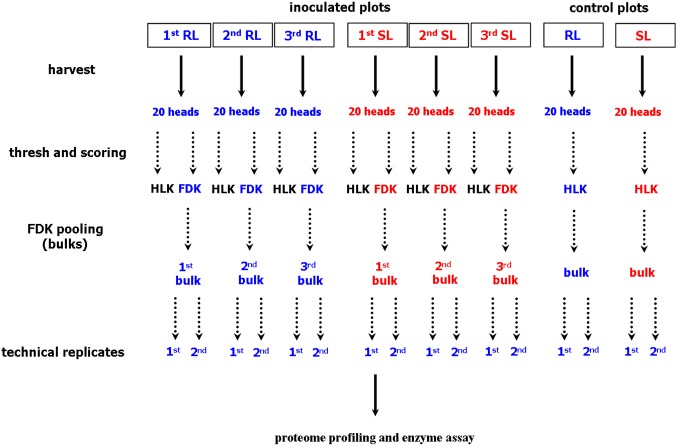
Diagram demonstrating a workflow of sample preparation for proteome analysis. Abbreviations: FDK, *Fusarium*-damaged kernels; HLK, healthy-looking kernels; RL, line of winter wheat more resistant to *Fusarium* head blight; SL, line of winter wheat more susceptible to *Fusarium* head blight.

The plant materials derived from one location (Cerekwica) were used for further molecular research – proteome profiling and alpha amylase activity assays. The FDK derived from 20 heads were pooled, separately for each inoculated plot, giving three separate pooled samples (bulks) for each analyzed line, the RL and the SL. The kernels derived from 20 heads of the control plot were also pooled for each analyzed line. The pooled samples (bulks) were used for proteomic research – each one in two technical replicates. Thus, the final proteomic survey resulted in 16 2-D gels. A diagram outlining the workflow of sample preparation for proteome analysis is shown in the Fig. 1.

The proteomic protocol used, including 2-DE and MS to identify differentially accumulated kernel proteins between the RL and SL of wheat, was the same as that described in detail by Masojć and Kosmala [31] and Masojć et al. [32]. Protein extraction was performed according to the method of Hurkman and Tanaka [33] using 0.7 M sucrose, 0.5 M TRIS, 30 mM HCl, 50 mM EDTA, 2% DTT, and 0.1 M KCl as components of the extraction buffer. In the first dimension, isoelectrofocusing (IEF), strip gels with linear pH range 4–7 (24 cm) were used to focus the aliquots of proteins extracted from 25 mg of wheat flour. In the second dimension (sodium dodecyl sulphate-polyacrylamide gel electrophoresis) the proteins were separated using 13% polyacrylamide gels (1.5×255×196 mm). Following electrophoresis the gels were stained with colloidal coomassie brilliant blue G-250, using the modified method of Neuhoff et al. [34]. The protein gels were scanned by Image scanner III (GE Healthcare, Buckinghamshire, UK) and subjected to Lab scan 6.0 program (GE Healthcare, Buckinghamshire, UK) processing. Spot detection and image analyses were performed with Image Master 2-D *Platinum* software (GE Healthcare, Buckinghamshire, UK). The abundance of each protein spot was normalized as a relative volume (% Vol) and it was calculated by Image Master software as a ratio of the volume of particular spot to the total volume of all the spots present on the gel. To consider a spot as ‘present’, it had to be detected in all the gel replicates used in the analysis. The significance of the differences was assessed using the Student’s t-test. The protein spots which showed at least two fold differences (p≤0.05) in protein abundance between the FDK of two analyzed lines together with protein spots present only in the FDK of one of the analyzed lines (the RL or SL), were subjected to MS analyses. However, to be sure that a given protein spot originated from wheat but not from *Fusarium*, one more condition had to be fulfilled by the spot. It had to be also present on the 2-D protein maps obtained for the control conditions, before inoculation.

Gel spots of interest were harvested, and after sequential washing with ammonium bicarbonate and acetonitrile were reduced with 10 mM dithiothreitol and alkylated with 55 mM iodoacetamide. The in-gel protein digestion was performed using trypsin solution (Sequencing Grade Modified Trypsin; Promega, Fitchburg, WI, USA) in 25 mM ammonium bicarbonate (25 ng µl^−1^). Samples were concentrated and desalted on a RP-C18 precolumn (Waters), and further peptide separation was achieved on a nano-Ultra Performance Liquid Chromatography (UPLC) RP-C18 column (Waters, BEH130 C18 column, 75 µl i.d., 250 mm long) of a nanoACQUITY UPLC system, using a 45 min linear acetonitrile gradient. The proteins were analyzed by liquid chromatography coupled to the Orbitrap Velos type mass spectrometer (Thermo), working in the regime of data dependent MS to MS/MS switch, in the Laboratory of Mass Spectrometry, Institute of Biochemistry and Biophysics, Polish Academy of Sciences (Warsaw, Poland) as described by Kosmala et al. [35] and Perlikowski et al. [36]. The data were analyzed with Mascot Distiller software (version 2.3, MatrixScience) with standard settings for the Orbitrap low resolution measurements (available at http://www.matrixscience.com/distiller.html) to extract MS/MS peak-lists from the raw files. The obtained fragmentation spectra were matched to the National Center Biotechnology Information (NCBI) non-redundant database (37425594 sequences; 13257553858 residues), with a *Viridiplantae* filter (1760563 sequences) using the Mascot search engine (Mascot Daemon v. 2.3.0, Mascot Server v. 2.4.0, MatrixScience). The following search parameters were applied: enzyme specificity was set to trypsin, peptide mass tolerance to ±40 ppm and fragment mass tolerance to ±0.8 Da. The protein mass was left as unrestricted and mass values as monoisotopic with one missed cleavage being allowed. Alkylation of cysteine by carbamidomethylation as fixed and oxidation of methionine as variable modifications, were set. Ion score was −10*Log(P), where P was the probability that the observed match was a random event. Only peptides with Mascot expect value over 0.05 were accepted as valid identifications. The proteins characterized by the highest Mascot-assigned protein score – Multidimensional Protein Identification Technology-type (MudPIT-type) and/or the highest number of peptide sequences, were selected. When the protein was identified as “a predicted protein” (primary identification), its amino acid sequence was blasted using *blastp* algorithm (http://blast.ncbi.nlm.nih.gov). The protein with the highest score was then selected as the functional homolog of the “predicted protein”.

### Alpha-amylase activity assays

Alpha-amylase activity in wheat kernels was evaluated according to the Ceralpha method [37] using Megazyme reagents (Ceralpha α-Amylase Assay Kit) and a detail protocol available at the company website: www.megazyme.com. The same biological and technical sample replicates as for proteome profiling, were applied (Fig. 1). Each technical replicate involved 0.5 g of wheat flour. The enzyme activity was expressed in Ceralpha Units (CU) per gram of flour – one unit of activity was defined as the amount of enzyme, in the presence of excess α-glucosidase and glucoamylase, required to release one micromole of *p*-nitrophenol from blocked *p*-nitrophenyl maltoheptaoside (BPNPG7) in one minute under the defined assay conditions. The significance of the differences in amylase activity between the RL and SL was assessed using the Student’s t-test (p≤0.05).

## Results and Discussion

### Field experiments

The two analyzed winter wheat lines differed significantly in their levels of resistance to FHB as manifested by the values of FHBi and % FDK (with respect to kernel weight and number), after *F. culmorum* inoculation in Cerekwica and Radzikow (Table 1). Thus, it is clearly visible that these two groups of plants (the RL and SL) could serve as excellent models to recognize the most crucial proteins associated with the resistance to FHB in winter wheat. In the control conditions (without the artificial inoculation) both lines, the RL and SL, demonstrated similar yield levels (Table 1). The differences observed between both locations might be due to different soil quality and weather conditions (Table S1).

**Table 1:** The components of the resistance to *Fusarium* head blight in the more resistant (RL) and more susceptible (SL) winter wheat (*Triticum aestivum*) lines and their yields under control conditions.

Location	Winterwheat line	Conditions after inoculation					Control conditions	
		FHBi	% FDK(weight [g])	% FDK(number)	Totalkernelnumber/head	Totalkernelweight [g]/head	Totalkernelnumber/head	Totalkernelweight [g]/head
Cerekwica	RL	23.7±0.58	45.5±5.14	54.9±5.70	27.9±4.23	0.7±0.25	39.9	2.0
	SL	38.3±2.08	83.5±4.89	91.1±4.90	16.6±1.89	0.3±0.06	38.4	1.9
Radzikow	RL	29.5±2.65	19.5±1.37	30.3±10.01	29.1±4.63	0.8±0.13	42.3	1.6
	SL	53.3±10.07	34.1±3.68	51.5±6.13	21.9±1.26	0.7±0.07	44.5	1.8

FHBi – *Fusarium* head blight index, FDK – *Fusarium*-damaged kernels, RL – more resistant line, SL – more susceptible line; mean values and standard deviations of each parameter calculated after inoculation (three plots) and data from one plot calculated for the control conditions, are shown.

### Proteome profiles and identities of differentially accumulated proteins

**Figure 2:**
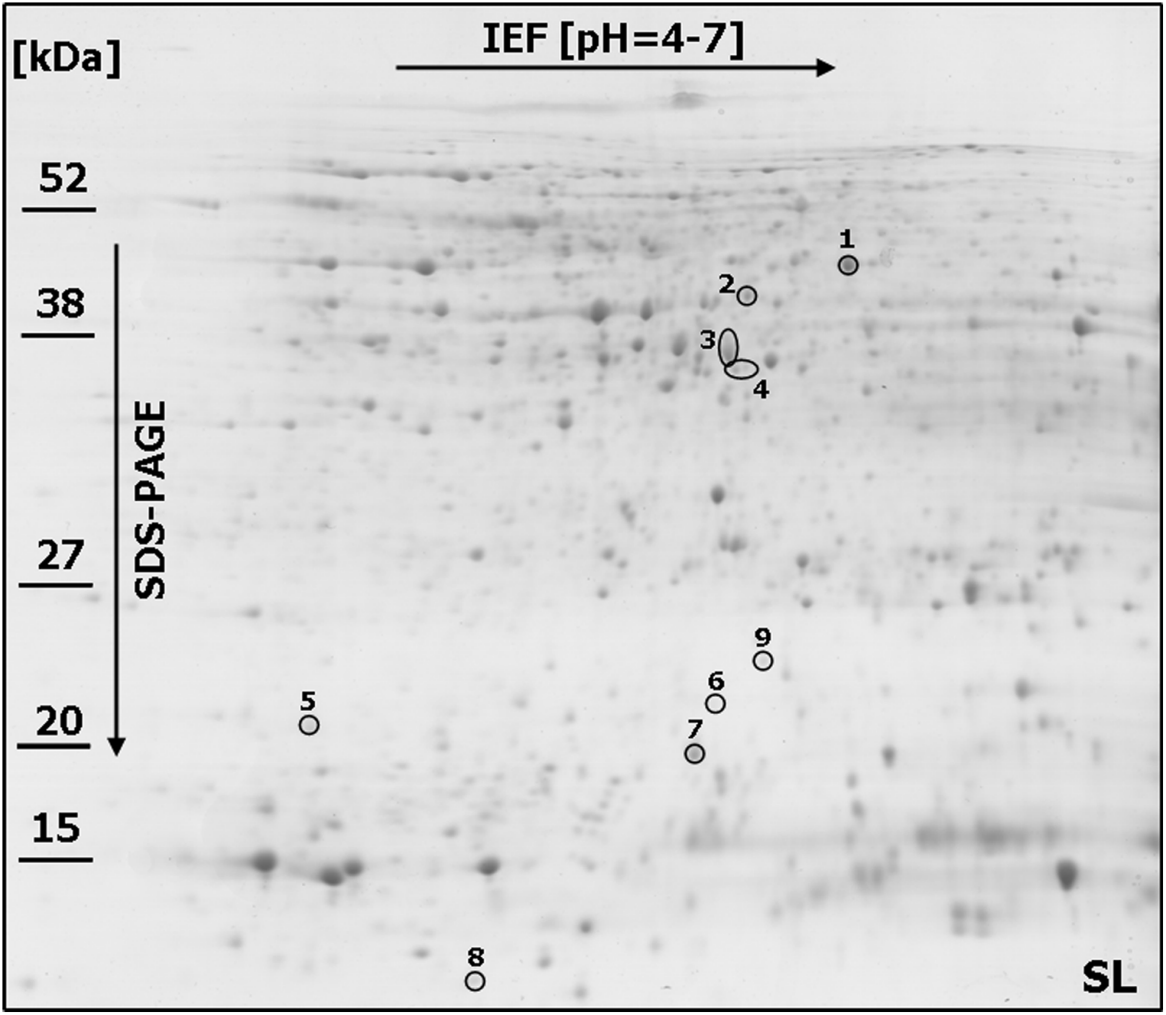
One representative 2-DE protein map of winter wheat (*Triticum aestivum*) kernel after *Fusarium culmorum* infection (*Fusarium*-damaged kernels) for the line more susceptible (SL) to *Fusarium* head blight. The spots with differentially accumulated (p≤0.05) proteins (1–9) identified in the SL, are circled with a solid line. Molecular weight (MW) scale is shown.

**Figure 3:**
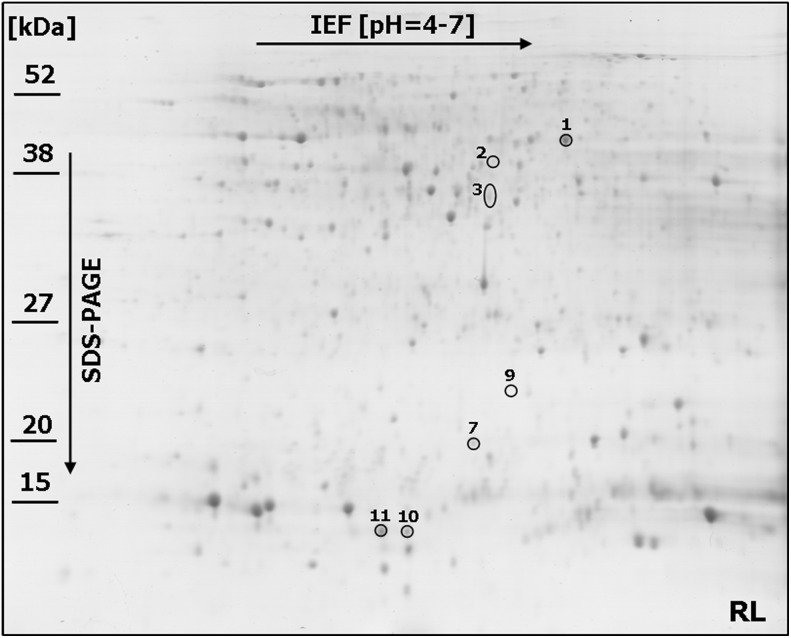
One representative 2-DE protein map of winter wheat (*Triticum aestivum*) kernel after *Fusarium culmorum* infection (*Fusarium*-damaged kernels) for the line more resistant (RL) to *Fusarium* head blight. The spots with differentially accumulated (p≤0.05) proteins (1–3, 7, 9–11) identified in the RL, are circled with a solid line. Molecular weight (MW) scale is shown.

**Figure 4:**
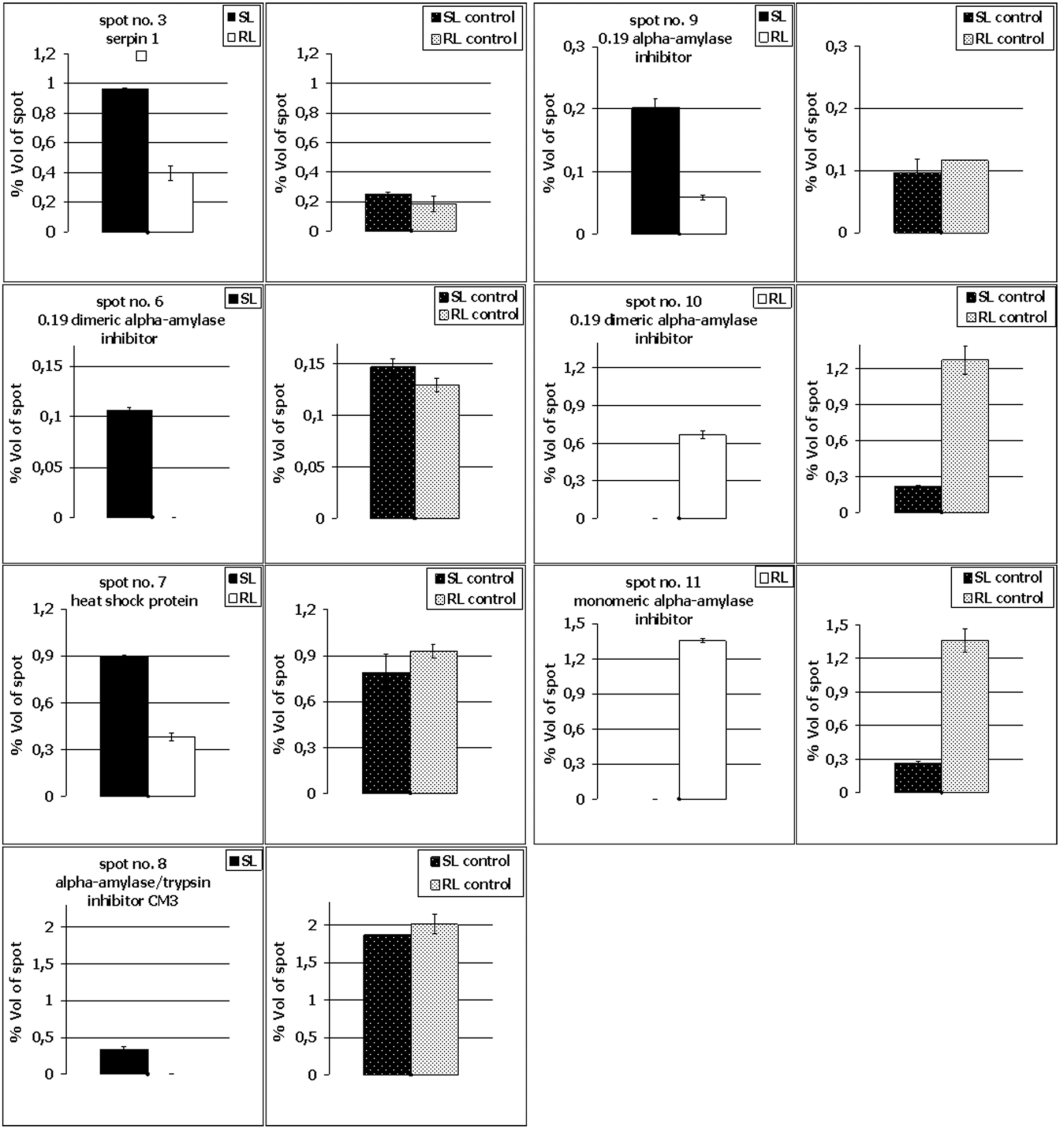
Comparison of selected kernel protein abundance after *Fusarium culmorum* infection and in the control conditions in the winter wheat (*Triticum aestivum*) SL (line more susceptible to *Fusarium* head blight) and the RL (line more resistant to *Fusarium* head blight). Spot numbering is the same as in Fig. 2, 3, S1 and S2. The standard deviation bars are shown. Only proteins identified from homogenous spots are shown.

Linear strips for isoelectrofocusing with pH 4–7 range were selected to resolve the proteins derived from FDK (after inoculation) and kernels (in the control conditions) of both analyzed wheat lines to achieve the best compromise between the number (656 protein spots in the RL and 658 in the SL were present within all the replicate gels) and resolution of the spots. Only the representative proteome maps were presented in the paper (Fig. 2 and 3; Fig. S1 and S2). All the analyzed maps showed a relatively high level of similarity with respect to the number of detected spots and their distribution in the gels. This advantage was, in our opinion, mainly due to the scientific approach applied into protein sample preparation – pooling of all the FDK into a single sample (bulk) for a given field plot. This strategy could help to identify the differences between the analyzed wheat lines, which could be associated with their level of resistance to FHB. On the other hand, the spot intensities cannot be compared directly between the gels as a single raw image is not suitable to reveal the protein abundance directly. For each spot the normalized volume of mean derived from gel replicates, to calculate the level of protein accumulation, was used here. The comparative analyses indicated a total of 11 spots that showed differences in protein abundance (according to the criteria described in the materials and methods section) between the more resistant and more susceptible wheat lines after infection (Fig. 2 and 3). Four protein spots (no. 4, 5, 6 and 8) were demonstrated to be specific for the SL (Fig. 2 and 4; Fig. S3) and two other protein spots (no. 10 and 11) for the RL (Fig. 3 and 4), although all these spots were observed in the RL and SL control gels (Fig. 4; Fig. S1 and S2). Five protein spots revealed quantitative differences between the analyzed lines, including four spots with significantly higher protein abundance in the more susceptible line (spots no. 2, 3, 7 and 9) and one spot with significantly higher abundance in the more resistant line (spot no. 1) (Fig. 4; Fig. S3). All the selected protein spots were subjected to mass spectrometry identification (Table 2). In some cases the selected proteins derived from wheat kernels were identified as homologues of proteins from related plant species (Table 2). For protein spot no. 1 no clear identification was found in the database. The spots no. 2, 4 and 5 were shown to be heterogeneous with more than one protein identified within them (Table 2). Thus, the abundance evaluation of particular proteins present in the spot was not possible and the total protein abundance was shown (Fig. S3). Approaches to deal with multi-protein spots would be required to determine relative abundance, for example, the spectral counting method described by Ishihama et al. [38]. The majority of spots were homogenous, containing one specific protein, and clear identifications were demonstrated for them.

**Table 2:** The results of MS analysis performed on the spots that showed at least a 2.0 ratio (p≤0.05) in protein abundance between the more resistant and more susceptible winter wheat (*Triticum aestivum*) lines (spots no. 1–3, 7 and 9), and spots present only in one line (spots no. 4–6, 8, 10 and 11).

Spot no.^1^	Accession^2^	Identified protein^3^	Score^4^	Coverage (%)^5^	No. of peptidematched	Theor. MW[kDa]/pI^6^	Exp. MW [kDa]/pI^7^	Abundance^8^
1	NP001141324	uncharacterized protein[*Zea mays*]	1993	59	22	38.3/5.4	46/6.2	2.0 fold higher in the RL
2*	EMT04083	LL-diaminopimelateaminotransferase,chloroplastic [*Aegilops* *tauschii*]	995	41	11	46.3/5.5	42/6.0	2.3 fold higher in the SL
	CAO77315	putative acyl transferase 4[*Triticum aestivum*]	890	37	13	46.8/5.7	42/6.0	2.3 fold higher in the SL
	AAB99745	HSP70 [*Triticum aestivum*]	835	20	10	71.4/5.1	42/6.0	2.3 fold higher in the SL
3	ACN59483	serpin 1 [*Triticum aestivum*]	896	29	10	43.3/5.4	37/5.9	2.4 fold higher in the SL
4*	BAK01819	predicted protein [*Hordeum* *vulgare* subsp. *vulgare*] *blastp:*Fructokinase-1 [*Oryza sativa*]	1152	50	14	34.9/5.7	36/6.0	present only in the SL
	EMS46550	putative NADP-dependentoxidoreductase P1 [*Triticum* *urartu*]	1014	37	12	38.4/5.5	36/6.0	present only in the SL
5*	BAK02140	predicted protein [*Hordeum* *vulgare* subsp. *vulgare*] *blastp:*peroxiredoxin-5 [*Zea mays*]	753	44	7	24.2/9.0	21/5.0	present only in the SL
	ABI54484	dimeric alpha-amylase inhibitor[*Triticum dicoccoides*]	654	69	6	13.7/6.5	21/5.0	present only in the SL
6	AAV39524	0.19 dimeric alpha-amylaseinhibitor [*Aegilops tauschii*]	631	67	7	13.9/7.5	22/5.9	present only in the SL
7	BAJ94129	predicted protein [*Hordeum* *vulgare* subsp. *vulgare*] *blastp:*heat shock protein [*Triticum* *aestivum*]	692	52	6	16.9/5.8	19/5.8	2.4 fold higher in the SL
8	P17314	alpha-amylase/trypsin inhibitorCM3 [*Triticum aestivum*]	492	47	5	18.9/7.4	11/5.4	present only in the SL
9	P01085	0.19 dimeric alpha-amylaseinhibitor [*Triticum aestivum*]	492	56	5	13.9/6.7	24/6.1	3.5 fold higher in the SL
10	AAV39517	0.19 dimeric alpha-amylaseinhibitor [*Triticum aestivum*]	760	89	8	13.8/5.7	13/5.6	present only in the RL
11	ABO45988	monomeric alpha-amylaseinhibitor [*Triticum aestivum*]	687	84	8	13.6/5.4	13/5.5	present only in the RL

1 Spot numbering was the same as in Fig. 2, 3, S1 and S2.

2 Database accession (according to NCBInr) of a homologous protein.

3 Homologous protein and organism from which it originates.

4 Mascot MudPIT (Multidimensional Protein Identification Technology) score.

5 Amino acid sequence coverage for the identified proteins (primary identifications); amino acid sequences for proteins (primary identifications) derived from the homogenous spots were shown in Fig. S4.

6 Theoretical molecular weight and isoelectric point revealed by Mascot software.

7 Experimental molecular weight and isoelectric point calculated based on 2-D protein maps.

8 Differences in accumulation level of proteins from the homogenous spots between the RL and SL after *Fusarium* infection. In case of heterogenous spots the same value for all the indicated proteins present in the spot, were shown. *heterogeneous spots with more than only one protein; the most abundant proteins were shown.

The 0.19 dimeric alpha-amylase inhibitor which significantly accumulated in the SL samples was identified in two spots – spot no. 6 and spot no. 9 (Fig. 4). These proteins might vary in post-translation modifications, resulting in different isoelectric points and molecular weights affecting spot positions in the 2-D gels (Fig. 2). The higher molecular weights of the inhibitor proteins identified in spots nos. 6 and 9, observed on 2-D maps (experimental values), compared to the theoretical values revealed after MS protein identification (Table 2) further suggest the presence of post-translation modifications to these proteins. The 0.19 dimeric alpha-amylase inhibitor was also found to highly accumulate in the RL (spot no. 10) (Fig. 4) and this protein differed by 11 amino acids with the isoform identified in the SL. It is highly probable that these two isoforms could possess quite different properties (Fig. S4).

Significant differences were observed in the amino acid sequences of the monomeric alpha-amylase inhibitor (spot no.11) and the 0.19 dimeric alpha-amylase inhibitor (spot no. 10), both of which highly accumulated in the RL (Fig. 4). A total of 38 mismatched amino acids and seven gaps were found (Fig. S4). Interestingly, both inhibitors (spot no. 10 and 11) likely represent the intact and functional proteins as almost no differences between their theoretical and experimentally evaluated molecular weights were observed (Table 2). A protein that slightly accumulated only in the SL was identified in spot no. 8 as the alpha-amylase/trypsin inhibitor CM3 (Fig. 4). Its higher theoretically evaluated molecular weight, compared to the experimentally calculated value, indicated partial degradation of this inhibitor (Table 2). Lastly, two further proteins identified as differentially accumulated between the RL and SL, and showing higher abundance in the more susceptible line were: serpin 1 (spot no. 3) and heat shock protein (spot no. 7) (Fig. 4).

### Potential involvement of the identified proteins in resistance to FHB

The proteins with higher abundance after *Fusarium* infection in the SL revealed no differences in the accumulation level between the analyzed lines in the control conditions (without infection) (Fig. 4). These proteins are probably not all closely associated with the development of resistance to FHB. For example, in this study the up-regulated serpin 1 was identified in the susceptible line (spot no. 3). Eggert et al. [28] reported a 90–225% induction of this protein in wheat after *F. graminearum* infection, when compared to the control kernels. Serpins belong to a group of proteins which are involved in the inhibition of serine proteases. Eggert et al. [28] suggested that the pathogen infection enhanced serpin accumulation, which might prevent the digestion of seed storage proteins caused by a fungal pathogen. The involvement of the identified serpin in the process of resistance to FHB is, however, unclear. It was shown earlier that serpins could also function as storage proteins when they lost their inhibitory activity [39]. It thus cannot be excluded that proteins highly accumulated in the SL might not be active enough to perform their function efficiently or they possess a relatively low activity. On the other hand, two proteins absent in the SL, monomeric alpha-amylase inhibitor (spot no.11) and 0.19 dimeric alpha-amylase inhibitor (spot no. 10), also had a lower accumulation level in this line during control conditions (Fig. 4 and Fig. S5), indicating lower resistance potential of the SL before infection.

### Inhibition of alpha-amylase activity could be a crucial component of the resistance to FHB in winter wheat

Starch is the main reserve compound accumulated in the endosperm of kernels comprising approximately 70% of kernel weight of wheat and the other cereals [40]. In the study of Jackowiak et al. [41] and Packa et al. [42] severe damage of starch granules leading to their complete disappearance in wheat and triticale kernels infected by *F. culmorum* were detected. Moreover, in the strongly infected kernels, the endosperm was replaced by mycelium. Alpha-amylase is a hydrolytic enzyme, which decomposes starch and makes carbohydrates available for germ development during sprouting, however, its activity is low in mature wheat kernels [43], [44]. *Fusarium* pathogens can use hydrolytic enzymes, including amylases to colonize kernels and acquire nitrogen and carbon from the endosperm [45]. The inoculation of wheat spikes with *F. culmorum* spores under field conditions showed increased alpha-amylase activity in kernels of different wheat species, including *T. monococum*, *T. dicoccum* and *T. aestivum* [46]. Thus, alpha-amylase inhibitors identified here in spots no. 10 and 11 could be involved in the development of resistance mechanisms and in the response to the synthesis of extracellular hydrolytic enzymes by infecting pathogens in the RL. The alpha-amylase inhibitors are thought to be important components of the active resistance of plants to necrotrophic pathogens [47].

**Figure 5:**
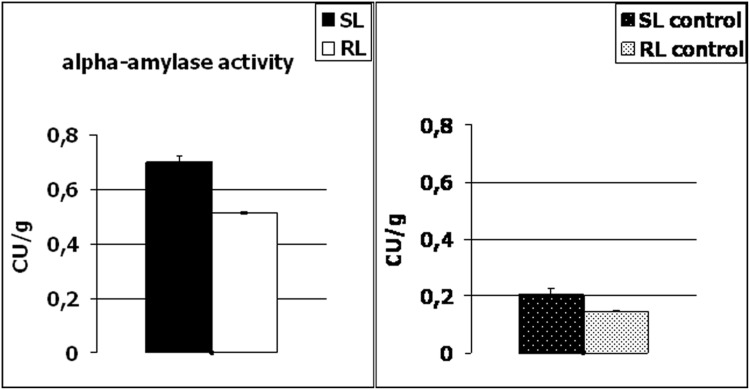
Comparison of alpha-amylase activity in the kernels of winter wheat (*Triticum aestivum*) SL (line more susceptible to *Fusarium* head blight) and RL (line more resistant to *Fusarium* head blight) after *Fusarium culmorum* infection (*Fusarium*-damaged kernels) and in control conditions. The enzyme activity was expressed in Ceralpha Units (CU) per gram of flour. The standard deviation bars are shown.

To verify the above hypothesis, alpha-amylase activity was determined here in the kernels of the RL and SL, after *F. culmorum* infection (in FDK) and in the control conditions (Fig. 5). From our results, it was clearly visible that the enzyme activity was lower in the control conditions in the both analyzed wheat lines and it increased significantly after inoculation. Thus, this could suggest that amylase is involved in the propagation of pathogen infection and expansion of *Fusarium* biomass in the wheat heads. As indicated in Table 1 number and weight of FDK (% FDK) in the heads of the susceptible line were significantly higher, and a total kernel number and weight after inoculation, significantly lower, compared to the resistant line, in two applied locations of the experimental plots. Although other components of wheat resistance to FHB were not analyzed here in detail, it is quite possible that *Fusarium* expansion was higher in the SL within the infected kernels (FDK). The amylase activity revealed lower level (p≤0.05) in the RL, both in the control conditions and after inoculation, and it is highly probable that it was due to the presence of monomeric alpha-amylase inhibitor (spot no.11) and dimeric alpha-amylase inhibitor (spot no. 10), both highly accumulated in the line with higher resistance to FHB.

### The proteome network potentially involved in the resistance to FHB in cereals

The FHB resistant or susceptible cereal genotypes were characterized by many proteins related to carbon metabolism and photosynthesis which were down-regulated, while the up-regulated proteins were involved in antioxidant, jasmonic acid and ethylene signaling pathways, phenylpropanoid biosynthesis, antimicrobial compound synthesis, detoxification, cell wall fortification, defense-related responses, amino acid synthesis and nitrogen metabolism [48]. On the other hand, susceptible genotypes likely reflected the delayed activation of the salicylic acid defense pathway [49]. Eggert and Pawelzik [27] studied the effect of *F. graminearum* and *F. culmorum* infection on the proteome of naked barley grains. The proteins up-regulated in the infected samples in comparison to controls belong to a protein group involved in regulation of transcription (e.g. three Dof zinc finger proteins, DNA-direct RNA polymerase). Up-regulated proteins identified were also: one NBS-LRR disease-resistance protein and three serpins with protease-inhibitor and nutritional-reservoir functions. Down-regulated proteins were connected to starch synthesis processes, protein synthesis inhibition and fungal chitin hydrolysis. Zhou et al. [29] identified up-regulated proteins with antioxidant functions (superoxide dismutase, dehydroascorbate reductase and glutathione S-transferase). The PR-2 protein (β-1, 3 glucanase) was also shown to be up-regulated. Zhang et al. [50] studied two near isogenic wheat lines (NILs), NIL75 (*Fhb1^+^*NIL) and NIL98 (*Fhb1* ^−^NIL), which were developed by backcrossing of ‘Clark’ (a highly FHB susceptible parent) to ‘Ning 7840’ (*Fhb1* donor) seven times (*Fhb1^+^*NIL contains less than 0.5% of donor genome) and identified proteins which were accumulated in the *Fhb1^+^*NIL, but not in the *Fhb1^−^*NIL, after *Fusarium* inoculation. These involved wheat proteins associated with defending fungal penetration (chitinases), photosynthesis (NAD(P)(+)-binding proteins, oxygen-evolving enhancer proteins) and energy metabolism (nucleoside diphosphate kinases).

## Conclusions

We proved that FDK of both, the more resistant and more susceptible winter wheat lines differed in their proteome profiles after *F. culmorum* infection under field conditions and these kernels were shown to be a suitable plant material to identify the crucial proteins potentially involved in the resistance to FHB. The advantage of the research presented here is the selection of two proteins from hundreds of protein spots observed on 2-D maps, with the impact on the FHB resistance in the analyzed wheat lines. It was mainly due to: (i) pooled samples (bulks) used for the analysis and (ii) protein spot selection. Only the spots which showed at least a two-fold difference in protein abundance between two analyzed wheat lines were subjected to MS analyses. Moreover, we put special emphasis on the proteins which showed high accumulation levels in the control conditions and are potentially involved in the development of resistance before infection (amylase inhibitors highly accumulated in the RL). Alpha-amylase activity assays revealed the highest level of enzyme activity in the line more susceptible to FHB after *F. culmorum* infection. Finally, we suggest that the inhibition of pathogen amylase activity could be one of the most crucial mechanisms to prevent infection progress in the analyzed resistant wheat line. In our opinion, the presented results are an important contribution to the field comprising proteomic network associated with FHB resistance in wheat, however, further work to identify other components is still required.

## Supporting Information

Figure S1 **One representative 2-DE protein map of winter wheat (** ***Triticum aestivum*** **) kernel without** ***Fusarium culmorum*** **infection (control conditions) for the line more susceptible (SL) to** ***Fusarium*** **head blight.** The spots with differentially accumulated (p≤0.05) proteins (1–11) identified in the SL and RL (line more resistant to *Fusarium* head blight) after infection, are circled with a solid line. Molecular weight (MW) scale is shown.
(TIF)

Figure S2 **One representative 2-DE protein map of winter wheat (** ***Triticum aestivum*** **) kernel without** ***Fusarium culmorum*** **infection (control conditions) for the line more resistant (RL) to** ***Fusarium*** **head blight.** The spots with differentially accumulated (p≤0.05) proteins (1–11) identified in the SL (line more susceptible to *Fusarium* head blight) and RL after infection, are circled with a solid line. Molecular weight (MW) scale is shown.
(TIF)

Figure S3 **Comparison of selected kernel protein abundance after** ***Fusarium culmorum*** **infection and in the control conditions in the winter wheat (** ***Triticum aestivum*** **) SL (line more susceptible to** ***Fusarium*** **head blight) and the RL (line more resistant to** ***Fusarium*** **head blight).** Spot numbering is the same as in the Fig. 2, 3, S1 and S2. The standard deviation bars are shown. Unidentified proteins and proteins derived from heterogeneous spots are shown.
(TIF)

Figure S4 **A. Amino acid sequences for proteins (primary identifications) derived from the homogenous spots.** In bold the amino acid sequence of peptides derived from winter wheat (*Triticum aestivum*), which were successfully matched to the protein sequences present in the database are indicated. Spot numbers, protein names and organism from which the protein originates are shown. B. Protein sequence alignment of alpha-amylase inhibitors identified in spots no. 6, 9, 10 and 11. C. Mascot search results for the identified proteins, including data for particular peptides.
(PDF)

Figure S5 **Enlarged windows with spots no. 10 and 11 selected in 2-DE gels of winter wheat (** ***Triticum aestivum*** **) kernel without** ***Fusarium culmorum*** **infection (control conditions) and after inoculation, for the line more resistant (RL) and more susceptible (SL) to** ***Fusarium*** **head blight.** The biological and technical replicates are shown.
(TIF)

Table S1 Meteorological conditions (sum of rainfalls and mean temperature) during the experiments performed in Cerekwica and Radzikow in 2013.
(PDF)
