# Supplementary figures and images for: Identification of Kernel Proteins Associated with the Resistance to Fusarium Head Blight in Winter Wheat (Triticum aestivum L.)
Source: PLoS One. 2014 Oct 23;9(10):e110822. doi: 10.1371/journal.pone.0110822 (PMC4207761; doi:10.1371/journal.pone.0110822)

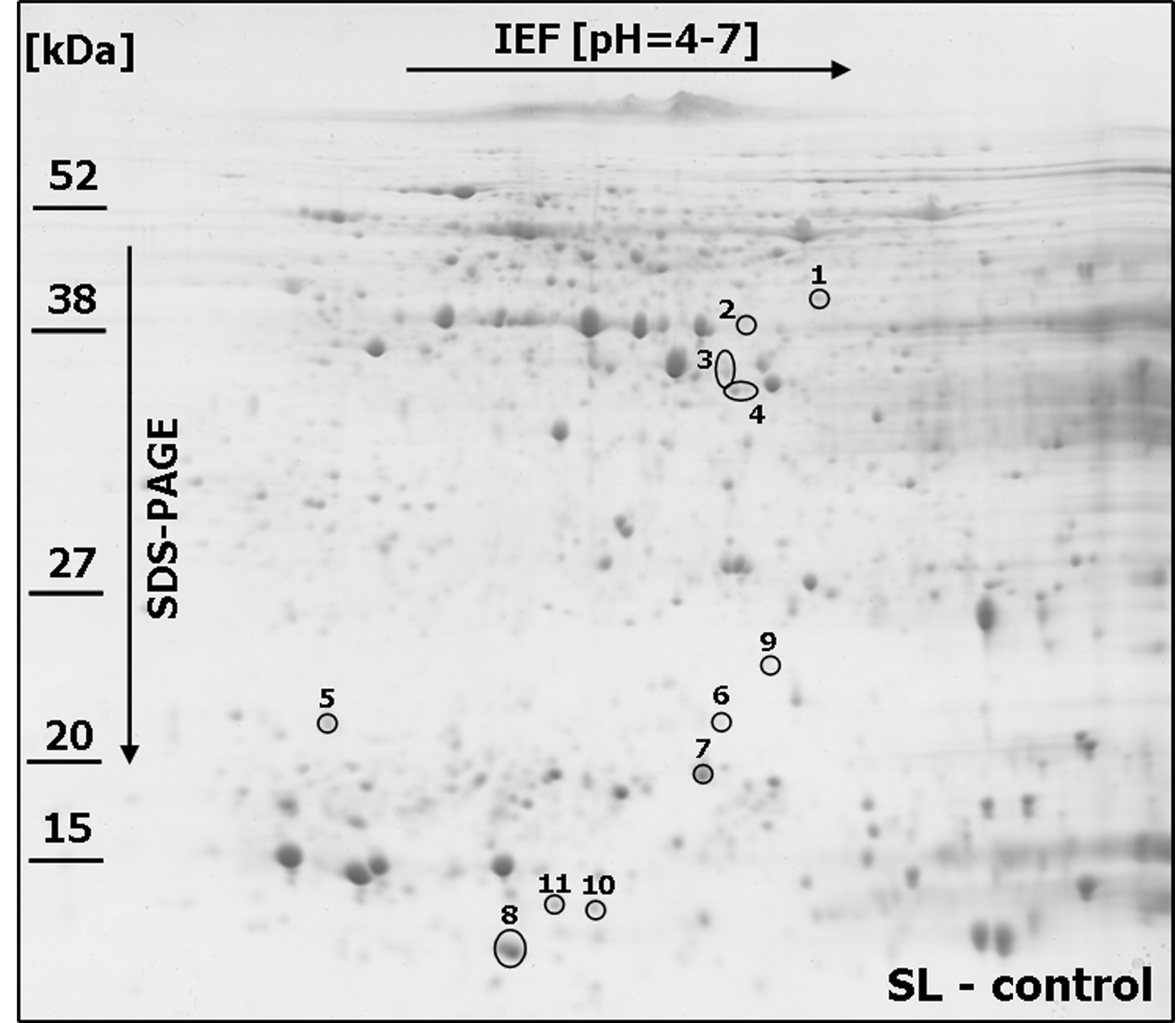

Supplement: Figure S1 — One representative 2-DE protein map of winter wheat ( Triticum aestivum ) kernel without Fusarium culmorum infection (control conditions) for the line more susceptible (SL) to Fusarium head blight. The spots with differentially accumulated (p≤0.05) proteins (1–11) identified in the SL and RL (line more resistant to Fusarium head blight) after infection, are circled with a solid line. Molecular weight (MW) scale is shown. (TIF) [file pone.0110822.s001.tif]

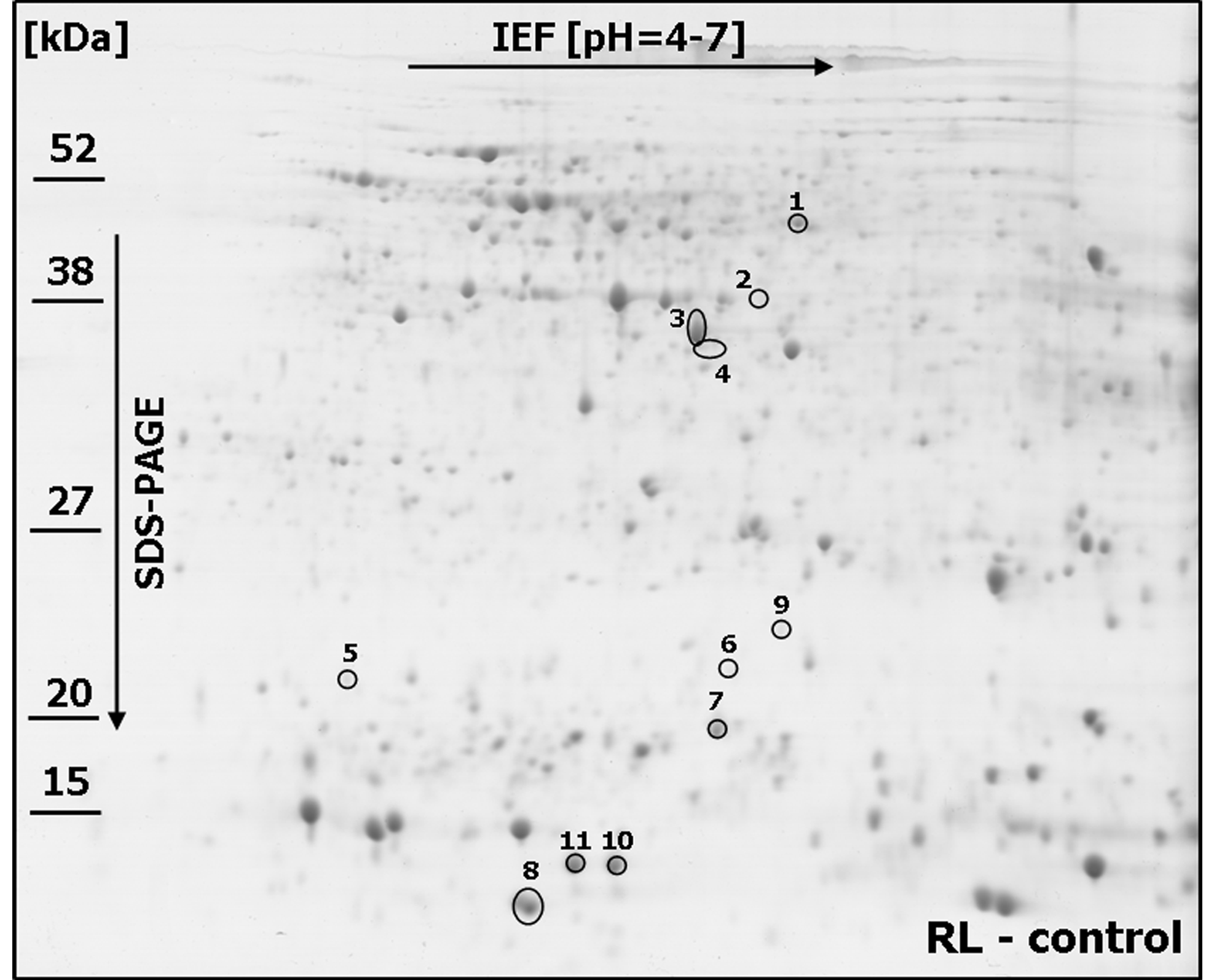

Supplement: Figure S2 — One representative 2-DE protein map of winter wheat ( Triticum aestivum ) kernel without Fusarium culmorum infection (control conditions) for the line more resistant (RL) to Fusarium head blight. The spots with differentially accumulated (p≤0.05) proteins (1–11) identified in the SL (line more susceptible to Fusarium head blight) and RL after infection, are circled with a solid line. Molecular weight (MW) scale is shown. (TIF) [file pone.0110822.s002.tif]

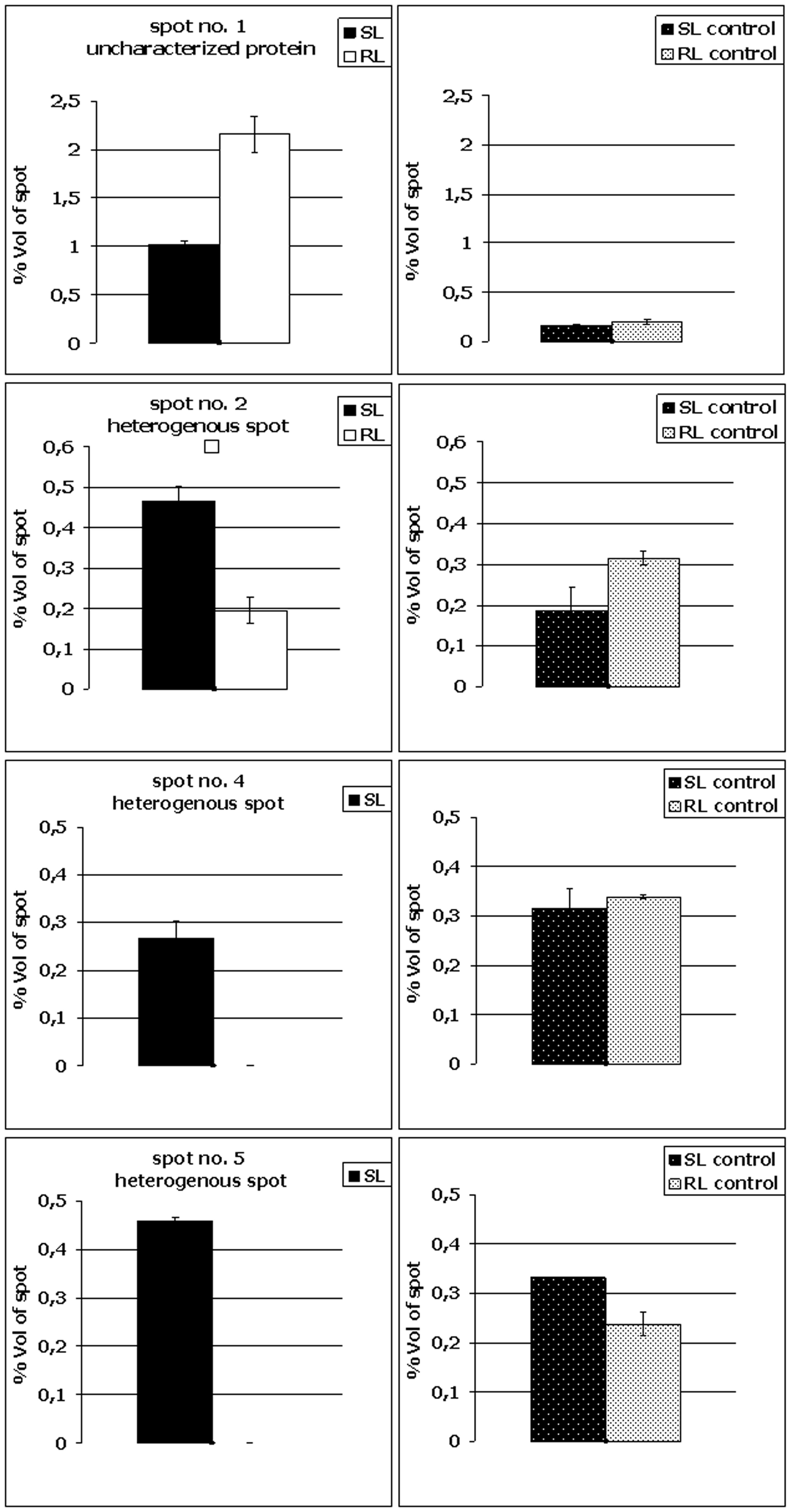

Supplement: Figure S3 — Comparison of selected kernel protein abundance after Fusarium culmorum infection and in the control conditions in the winter wheat ( Triticum aestivum ) SL (line more susceptible to Fusarium head blight) and the RL (line more resistant to Fusarium head blight). Spot numbering is the same as in the Fig. 2, 3, S1 and S2. The standard deviation bars are shown. Unidentified proteins and proteins derived from heterogeneous spots are shown. (TIF) [file pone.0110822.s003.tif]

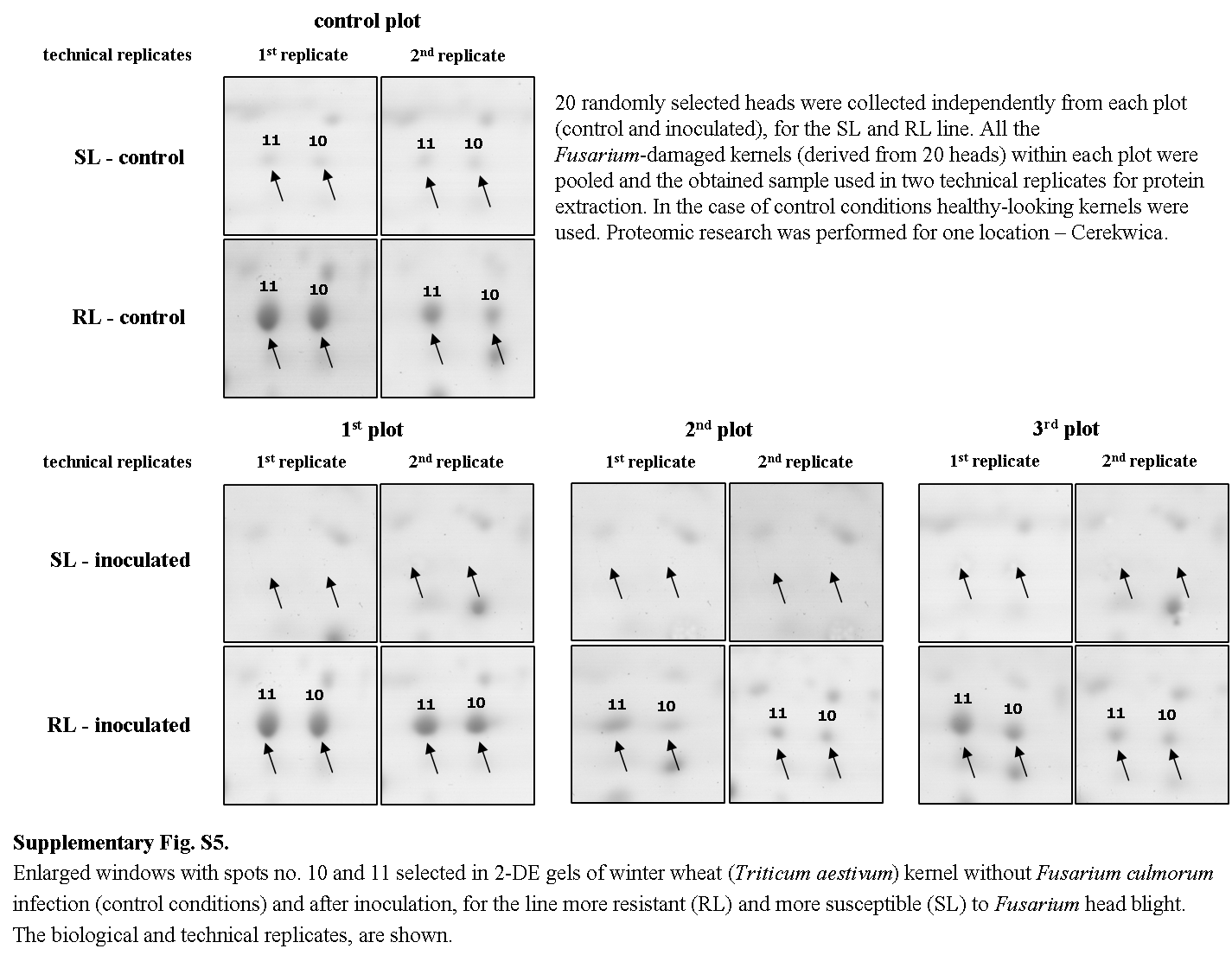

Supplement: Figure S5 — Enlarged windows with spots no. 10 and 11 selected in 2-DE gels of winter wheat ( Triticum aestivum ) kernel without Fusarium culmorum infection (control conditions) and after inoculation, for the line more resistant (RL) and more susceptible (SL) to Fusarium head blight. The biological and technical replicates are shown. (TIF) [file pone.0110822.s005.tif]
