# Supplementary material for: Identification of Kernel Proteins Associated with the Resistance to Fusarium Head Blight in Winter Wheat (Triticum aestivum L.)
Source: PLoS One. 2014 Oct 23;9(10):e110822. doi: 10.1371/journal.pone.0110822 (PMC4207761; doi:10.1371/journal.pone.0110822)
Supplement: Figure S4 — A. Amino acid sequences for proteins (primary identifications) derived from the homogenous spots. In bold the amino acid sequence of peptides derived from winter wheat (Triticum aestivum), which were successfully matched to the protein sequences present in the database are indicated. Spot numbers, protein names and organism from which the protein originates are shown. B. Protein sequence alignment of alpha-amylase inhibitors identified in spots no. 6, 9, 10 and 11. C. Mascot search results for the identified proteins, including data for particular peptides. (PDF) [file pone.0110822.s004.pdf]

Supplementary Fig. S4.

A. Amino acid sequences for proteins (primary identifications) derived from the homogenous spots. In bold the amino acid sequence of peptides derived from winter wheat (*Triticum aestivum*), which were successfully matched to the protein sequences present in database, are indicated. Spot numbers, protein names and organism from which the protein originates are shown.

Spot. 1. uncharacterized protein [*Zea mays*]

1 **MSHLGRPNGS PNEK**YSLKPV VPELEKLLGK **KVTFAPDCVG PEVEEIVNK**A

51 EDGAVILLEN LRFHIEEEGS CKDKEGNKTK ADKAQVEAFR **KGLTALGDVY**

101 **INDAFGTAHR AHSSMVGVDL PQKASGFLVK KELEYFAK**AL EEPKRPFLAI

151 LGGAKVSDK**I QLIDNLLDKV NTLIICGGMA FTFK**KTLEGV SIGNSLFDEA

201 GSKTVGNLVE KAKAKGVK**LV LPVDYITADK FDKDANTGYA TDKDGIPDGW**

251 **QGLDCGEESV K**LYKEAIAEA QTILWNGPAG VFEFEKFASG TK**ATLDAVVD**

301 **AVQK**DGK**IVI IGGGDTATVA KKYGVEDKLS HVSTGGGASL ELLEGKELPG**

351 **VTALSSK**

Spot. 2. heterogenous spot

Spot. 3. serpin 1 [*Triticum aestivum*]

1 MATTLATDVR **LSIAHQTR**FA LR**LASTISSN PK**SAASNAAF SPVSLHSALS

51 LLAAGAGSAT RDQLVATLGT GEVEGLHALA EQVVQFVLAD ASSAGGPRVA

101 FANGVFVDAS LLLKPSFQEL AVCK**YKAETQ SVDFQTKAAE VTTQVNSWVE**

151 **K**VTSGRIK**NI LPSGSVDNTT KLVLANALYF K**GAWTDQFDS YGTKNDYFYL

201 LDGSSVQTPF MSSMDDDQYI SSSDGLKVLK LPYKQGGDNR QFSMYILLPE

251 APGCLSSLAE K**LSAEPDFLE R**HIPRQRVAI RQFKLPKFK**I SFGIEASDLL**

301 **K**CLGLQLPFS DEADFSEMVD SPMPQGLR**VS SVFHQAFVEV NEQGTEAAAS**

351 **TAIK**MVPQQA RPPSVMDFIA DHPFLFLLRE DISGVVLFMG HVVNPLLSS

Spot. 4. heterogenous spot

Spot. 5. heterogenous spot

Spot. 6. 0.19 dimeric alpha-amylase inhibitor [*Aegilops tauschii*]

1 SGPWMCYPGQ AFQVPALPAC RPLLR**LQCNG SQVPEAVLRD CCQQLAHISE**

51 **WCRCGALYSM LDSMYKXHGA QEGQAGTGAF PR**CRREVVK**L TAASITAVCR**

101 **LPIVVDASGD GAYVCK**DVAA YPDA

Spot. 7. predicted protein [*Hordeum vulgare subsp. vulgare*]

1 MSIVRR**SNVF DPFADLWADP FDTFRSIVPA ISGGNSETAA FANAR**MDWKE

51 TPEAHVFKAD LPGVKK**EEVK VEVEDGNVLV VSGER**TKEKE DKNDKWHRME

101 RSSGKFVRR**F RLPEDAK**VEE VK**AGLENGVL TVTVPK**AEVK KPEVKAIEIS
151 G

Spot. 8. alpha-amylase/trypsin inhibitor CM3 [*Triticum aestivum*]

1 MACKSSCSLL LLAAVLLSVL AAASASGSCV PGVAFRTNLL PHCR**DYVLQQ**
51 **TCGTFTPGSK** LPEWMTSASI YSPGKPYLAK **LYCCQELAEI SQQCR**CEALR
101 **YFIALPVPSQ PVDPRSGNVG ESGLIDLPGC PR**EMQWDFVR **LLVAPGQCNL**
151 **ATIHNVR**YCP AVEQPLWI

Spot. 9. alpha-amylase inhibitor 0.19 [*Triticum aestivum*]

1 SGPWMCYPGQ AFQVPALPAC RPLLR**LQCNG SQVPEAVLR**D CCQQLAHISE
51 WCR**CGALYSM LDSMYKEHGA QEGQAGTGAF PR**CRREVVK**L TAASITAVCR**
101 **LPIVVDASGD GAYVCK**DVAA YPDA

Spot. 10. 0.19 dimeric alpha-amylase inhibitor [*Triticum aestivum*]

1 **SGPWMCYPGY AFKVPALPGC RPVLKLQCNG SQVPEAVLRE CCQQLADISE**
51 **WCRCGALYSM LDSMYKEHGV QEGQAGTGAF PSCR**REVVK**L TAASITAVCK**
101 **LPIVIDASGD GAYVCK**DVAA YPDA

Spot. 11. monomeric alpha-amylase inhibitor [*Triticum aestivum*]

1 **SGPWSWCDPA TGYKVSALTG CR**AMVK**LQCV GSQVPEAVLR DCCQQLADIN**
51 **NEWCRCGDLS SMLRSVYQEL GVR**EGKEVLP GCRKEVMK**LT AASVPEVCKV**
101 **PIPNPSGDGA GVCYWAAYPD V**

B. Protein sequence alignment of alpha-amylase inhibitors identified in spots no. 6, 9, 10 and 11 (amino acids different to those found in spot 6 and 9, were marked in red; 'x' – unknown amino acid; '-' – a gap).

Spot 6 1 SGPW-MCYPGQAFQVPALPACRPLLRLQCNGSQVPEAVLRDCCQQLAHI-SEWCRCGALY 58
Spot 9 1 SGPW-MCYPGQAFQVPALPACRPLLRLQCNGSQVPEAVLRDCCQQLAHI-SEWCRCGALY 58
Spot 10 1 SGPW-MCYPGYAFKVPALPGCRPVLKLQCNGSQVPEAVLRECCQQLADI-SEWCRCGALY 58
Spot 11 1 SGPWSWCDPATGYKVSALTGCRAMVKLQCVGSQVPEAVLRDCCQQLADINNEWCRCGDLS 60

Spot 6 59 SMLDSMYKXHGAQEGQAGTGAFPRCRREVVKLTAASITAVCRLPIVVDASGDGAYVCKDV 118
Spot 9 59 SMLDSMYKEHGAQEGQAGTGAFPRCRREVVKLTAASITAVCRLPIVVDASGDGAYVCKDV 118
Spot 10 59 SMLDSMYKEHGVQEGQAGTGAFPSCRREVVKLTAASITAVCKLPIVIDASGDGAYVCKDV 118
Spot 11 61 SMLRSVYQELGVREGKE---VLPGCRKEVMKLTAASVPEVCKVPI-PNPSGDGAGVCY-W 115

Spot 6 119 AAYPD 123

Spot 9 119 AAYPD 123

Spot 10 119 AAYPD 123

Spot 11 116 AAYPD 120

C. Mascot raw search results for the identified proteins, including data for particular peptides.

Spot no. 1
Ions score is -10*Log(P), where P is the probability that the observed match is a random event. Individual ions scores > 44 indicate identity or extensive homology (p<0.05). Protein scores are derived from ions scores as a non-probabilistic basis for ranking protein hits.

uncharacterized protein [*Zea mays*]

Query	Start – End	Observed	Mr (expt)	Mr (calc)	ppm	M	Score	Expect	Rank	U	Peptide
1121	1 – 14	762.3736	1522.7327	1522.7310	1.13	0	48	0.026	1	U	-.MSHLGRPNGSPNEK.Y
996	2 – 14	696.8536	1391.6926	1391.6906	1.46	0	68	0.00031	1	U	M.SHLGRPNGSPNEK.Y
1525	31 – 49	1066.0383	2130.0620	2130.0667	-2.19	1	121	1.1e-09	1	U	K.KVTFAPDCVGPEVEEIVNK.A
1526	31 – 49	711.0281	2130.0623	2130.0667	-2.06	1	72	8.1e-05	1	U	K.KVTFAPDCVGPEVEEIVNK.A
1527	31 – 49	711.0281	2130.0624	2130.0667	-2.05	1	55	0.0039	1	U	K.KVTFAPDCVGPEVEEIVNK.A
1529	31 – 49	711.0290	2130.0650	2130.0667	-0.79	1	56	0.0032	1	U	K.KVTFAPDCVGPEVEEIVNK.A
1457	32 – 49	1001.9827	2001.9508	2001.9718	-10.4	0	90	1.1e-06	1	U	K.VTFAPDCVGPEVEEIVNK.A
1458	32 – 49	1001.9866	2001.9586	2001.9718	-6.55	0	104	4.9e-08	1	U	K.VTFAPDCVGPEVEEIVNK.A
1459	32 – 49	1001.9867	2001.9588	2001.9718	-6.45	0	93	6.7e-07	1	U	K.VTFAPDCVGPEVEEIVNK.A
1460	32 – 49	1001.9899	2001.9652	2001.9718	-3.25	0	115	4.3e-09	1	U	K.VTFAPDCVGPEVEEIVNK.A
1461	32 – 49	668.3293	2001.9662	2001.9718	-2.79	0	45	0.043	1	U	K.VTFAPDCVGPEVEEIVNK.A
1462	32 – 49	1001.9908	2001.9670	2001.9718	-2.35	0	132	8.3e-11	1	U	K.VTFAPDCVGPEVEEIVNK.A
1463	32 – 49	668.3299	2001.9680	2001.9718	-1.88	0	87	2.4e-06	1	U	K.VTFAPDCVGPEVEEIVNK.A
1464	32 – 49	1001.9923	2001.9700	2001.9718	-0.85	0	89	1.5e-06	1	U	K.VTFAPDCVGPEVEEIVNK.A
1465	32 – 49	1001.9928	2001.9710	2001.9718	-0.35	0	99	1.5e-07	1	U	K.VTFAPDCVGPEVEEIVNK.A
1466	32 – 49	1001.9936	2001.9726	2001.9718	0.45	0	114	5.3e-09	1	U	K.VTFAPDCVGPEVEEIVNK.A
1467	32 – 49	1001.9940	2001.9734	2001.9718	0.85	0	95	4e-07	1	U	K.VTFAPDCVGPEVEEIVNK.A
1468	32 – 49	1001.9941	2001.9736	2001.9718	0.95	0	90	1.4e-06	1	U	K.VTFAPDCVGPEVEEIVNK.A
1469	32 – 49	1001.9944	2001.9742	2001.9718	1.24	0	61	0.0011	1	U	K.VTFAPDCVGPEVEEIVNK.A
1470	32 – 49	1001.9951	2001.9756	2001.9718	1.94	0	98	2.2e-07	1	U	K.VTFAPDCVGPEVEEIVNK.A
1471	32 – 49	1001.9951	2001.9756	2001.9718	1.94	0	75	4e-05	1	U	K.VTFAPDCVGPEVEEIVNK.A
1472	32 – 49	1001.9967	2001.9788	2001.9718	3.54	0	98	2.2e-07	1	U	K.VTFAPDCVGPEVEEIVNK.A
1516	91 – 110	530.5268	2118.0782	2118.0858	-3.60	1	48	0.021	1	U	R.KGLTALGDVYINDAFGTAHR.A
1517	91 – 110	707.0363	2118.0870	2118.0858	0.56	1	69	0.00015	1	U	R.KGLTALGDVYINDAFGTAHR.A
1436	92 – 110	664.3346	1989.9819	1989.9908	-4.50	0	103	7.7e-08	1	U	K.GLTALGDVYINDAFGTAHR.A

Query	Start – End		Observed	Mr (expt)	Mr (calc)	ppm	M	Score	Expect	Rank	U	Peptide
1437	92	– 110	664.3357	1989.9852	1989.9908	-2.82	0	103	7.8e-08	1	U	K.GLTALGDVYINDAFGTAHR.A
1438	92	– 110	664.3357	1989.9854	1989.9908	-2.76	0	106	3.9e-08	1	U	K.GLTALGDVYINDAFGTAHR.A
1439	92	– 110	664.3359	1989.9858	1989.9908	-2.56	0	85	4.5e-06	1	U	K.GLTALGDVYINDAFGTAHR.A
1440	92	– 110	664.3365	1989.9878	1989.9908	-1.53	0	90	1.4e-06	1	U	K.GLTALGDVYINDAFGTAHR.A
1441	92	– 110	996.0014	1989.9883	1989.9908	-1.26	0	120	1.3e-09	1	U	K.GLTALGDVYINDAFGTAHR.A
1442	92	– 110	996.0015	1989.9884	1989.9908	-1.23	0	125	4.5e-10	1	U	K.GLTALGDVYINDAFGTAHR.A
952	111	– 123	684.8485	1367.6824	1367.6867	-3.15	0	85	5.2e-06	1	U	R.AHSSMVGVDLPQK.A
953	111	– 123	456.9014	1367.6825	1367.6867	-3.10	0	46	0.036	1	U	R.AHSSMVGVDLPQK.A
977	111	– 123	692.8438	1383.6730	1383.6817	-6.28	0	55	0.0042	1	U	R.AHSSMVGVDLPQK.A + Oxidation (M)
979	111	– 123	462.2329	1383.6768	1383.6817	-3.50	0	66	0.00038	1	U	R.AHSSMVGVDLPQK.A + Oxidation (M)
980	111	– 123	692.8459	1383.6772	1383.6817	-3.21	0	51	0.011	1	U	R.AHSSMVGVDLPQK.A + Oxidation (M)
981	111	– 123	692.8460	1383.6775	1383.6817	-2.98	0	68	0.00026	1	U	R.AHSSMVGVDLPQK.A + Oxidation (M)
13	124	– 130	361.2147	720.4148	720.4170	-3.07	0	45	0.047	1	U	K.ASGFLVK.K
429	131	– 138	514.2749	1026.5351	1026.5386	-3.32	1	70	0.00013	1	U	K.KELEYFAK.A
688	160	– 169	592.8449	1183.6752	1183.6812	-5.07	0	72	9.2e-05	1	U	K.IQLIDNLLDK.V
690	160	– 169	592.8468	1183.6789	1183.6812	-1.91	0	50	0.013	1	U	K.IQLIDNLLDK.V
691	160	– 169	592.8468	1183.6791	1183.6812	-1.78	0	59	0.0015	1	U	K.IQLIDNLLDK.V
692	160	– 169	592.8471	1183.6796	1183.6812	-1.32	0	68	0.00019	1	U	K.IQLIDNLLDK.V
1192	170	– 184	836.4293	1670.8441	1670.8524	-4.99	0	68	0.00024	1	U	K.VNTLIICGGMAFTFK.K
1193	170	– 184	836.4308	1670.8471	1670.8524	-3.20	0	90	1.5e-06	1	U	K.VNTLIICGGMAFTFK.K
1194	170	– 184	836.4334	1670.8522	1670.8524	-0.16	0	75	4.6e-05	1	U	K.VNTLIICGGMAFTFK.K
1204	170	– 184	844.4285	1686.8424	1686.8474	-2.92	0	99	1.9e-07	1	U	K.VNTLIICGGMAFTFK.K + Oxidation (M)
1205	170	– 184	844.4297	1686.8449	1686.8474	-1.44	0	102	9e-08	1	U	K.VNTLIICGGMAFTFK.K + Oxidation (M)
922	219	– 230	673.8794	1345.7443	1345.7493	-3.75	0	45	0.049	1	U	K.LVLPVDYITADK.F
1241	219	– 233	868.9730	1735.9315	1735.9396	-4.68	1	49	0.016	1	U	K.LVLPVDYITADKFDK.D
1242	219	– 233	868.9738	1735.9331	1735.9396	-3.78	1	105	3.4e-08	1	U	K.LVLPVDYITADKFDK.D
1243	219	– 233	868.9745	1735.9344	1735.9396	-3.03	1	85	3.5e-06	1	U	K.LVLPVDYITADKFDK.D
1249	219	– 233	868.9762	1735.9379	1735.9396	-1.00	1	73	5e-05	1	U	K.LVLPVDYITADKFDK.D
1250	219	– 233	868.9764	1735.9382	1735.9396	-0.81	1	56	0.0029	1	U	K.LVLPVDYITADKFDK.D
1252	219	– 233	868.9769	1735.9393	1735.9396	-0.18	1	91	7.6e-07	1	U	K.LVLPVDYITADKFDK.D
1254	219	– 233	868.9784	1735.9423	1735.9396	1.53	1	58	0.0017	1	U	K.LVLPVDYITADKFDK.D
1255	219	– 233	868.9810	1735.9475	1735.9396	4.53	1	81	6.8e-06	1	U	K.LVLPVDYITADKFDK.D
1067	231	– 243	723.3303	1444.6461	1444.6470	-0.63	1	111	7.2e-09	1	U	K.FDKDANTGYATDK.D

Query	Start	– End	Observed	Mr (expt)	Mr (calc)	ppm	M	Score	Expect	Rank	U	Peptide
1068	231	– 243	482.5560	1444.6462	1444.6470	-0.60	1	47	0.017	1	U	K.FDKDANTGYATDK.D
466	234	– 243	528.2354	1054.4561	1054.4567	-0.53	0	62	0.00043	1	U	K.DANTGYATDK.D
467	234	– 243	528.2355	1054.4565	1054.4567	-0.17	0	68	9.9e-05	1	U	K.DANTGYATDK.D
1648	234	– 261	1000.1003	2997.2791	2997.2934	-4.79	1	117	6e-10	1	U	K.DANTGYATDKDGIPDGWQGLDCGEESVK.L
1649	234	– 261	1000.1027	2997.2863	2997.2934	-2.39	1	97	6.4e-08	1	U	K.DANTGYATDKDGIPDGWQGLDCGEESVK.L
1650	234	– 261	1000.1031	2997.2875	2997.2934	-1.99	1	134	1.3e-11	1	U	K.DANTGYATDKDGIPDGWQGLDCGEESVK.L
1651	234	– 261	1499.6543	2997.2940	2997.2934	0.20	1	104	1.3e-08	1	U	K.DANTGYATDKDGIPDGWQGLDCGEESVK.L
1652	234	– 261	1000.1072	2997.2998	2997.2934	2.11	1	69	4.8e-05	1	U	K.DANTGYATDKDGIPDGWQGLDCGEESVK.L
1653	234	– 261	1000.1088	2997.3046	2997.2934	3.71	1	66	9.5e-05	1	U	K.DANTGYATDKDGIPDGWQGLDCGEESVK.L
1399	244	– 261	981.4249	1960.8353	1960.8473	-6.09	0	102	2.7e-08	1	U	K.DGIPDGWQGLDCGEESVK.L
1400	244	– 261	981.4251	1960.8356	1960.8473	-5.96	0	66	0.00011	1	U	K.DGIPDGWQGLDCGEESVK.L
1401	244	– 261	981.4265	1960.8383	1960.8473	-4.56	0	111	3.6e-09	1	U	K.DGIPDGWQGLDCGEESVK.L
1402	244	– 261	981.4265	1960.8384	1960.8473	-4.53	0	52	0.0028	1	U	K.DGIPDGWQGLDCGEESVK.L
1403	244	– 261	981.4267	1960.8388	1960.8473	-4.33	0	96	1.2e-07	1	U	K.DGIPDGWQGLDCGEESVK.L
1404	244	– 261	981.4272	1960.8399	1960.8473	-3.75	0	97	1e-07	1	U	K.DGIPDGWQGLDCGEESVK.L
1405	244	– 261	981.4283	1960.8420	1960.8473	-2.67	0	128	7.2e-11	1	U	K.DGIPDGWQGLDCGEESVK.L
1406	244	– 261	654.6217	1960.8432	1960.8473	-2.06	0	80	5.2e-06	1	U	K.DGIPDGWQGLDCGEESVK.L
1407	244	– 261	981.4291	1960.8437	1960.8473	-1.82	0	128	7.7e-11	1	U	K.DGIPDGWQGLDCGEESVK.L
1408	244	– 261	981.4295	1960.8444	1960.8473	-1.48	0	128	7.8e-11	1	U	K.DGIPDGWQGLDCGEESVK.L
1409	244	– 261	981.4298	1960.8451	1960.8473	-1.11	0	78	7.8e-06	1	U	K.DGIPDGWQGLDCGEESVK.L
1410	244	– 261	981.4302	1960.8458	1960.8473	-0.78	0	45	0.016	1	U	K.DGIPDGWQGLDCGEESVK.L
1411	244	– 261	981.4306	1960.8466	1960.8473	-0.36	0	72	3.3e-05	1	U	K.DGIPDGWQGLDCGEESVK.L
1412	244	– 261	981.4314	1960.8482	1960.8473	0.46	0	98	7.9e-08	1	U	K.DGIPDGWQGLDCGEESVK.L
742	293	– 304	615.3364	1228.6582	1228.6663	-6.59	0	84	8.2e-06	1	U	K.ATLDAVVDAVQK.D
743	293	– 304	615.3375	1228.6605	1228.6663	-4.75	0	97	3.7e-07	1	U	K.ATLDAVVDAVQK.D
744	293	– 304	615.3377	1228.6608	1228.6663	-4.49	0	100	1.9e-07	1	U	K.ATLDAVVDAVQK.D
745	293	– 304	615.3378	1228.6611	1228.6663	-4.24	0	68	0.00034	1	U	K.ATLDAVVDAVQK.D
746	293	– 304	615.3379	1228.6612	1228.6663	-4.18	0	61	0.0017	1	U	K.ATLDAVVDAVQK.D
747	293	– 304	615.3379	1228.6613	1228.6663	-4.10	0	93	9.2e-07	1	U	K.ATLDAVVDAVQK.D
748	293	– 304	615.3379	1228.6613	1228.6663	-4.08	0	90	1.8e-06	1	U	K.ATLDAVVDAVQK.D
749	293	– 304	615.3381	1228.6616	1228.6663	-3.84	0	102	1.3e-07	1	U	K.ATLDAVVDAVQK.D
750	293	– 304	615.3382	1228.6619	1228.6663	-3.61	0	110	2.1e-08	1	U	K.ATLDAVVDAVQK.D
751	293	– 304	615.3383	1228.6621	1228.6663	-3.41	0	89	2.3e-06	1	U	K.ATLDAVVDAVQK.D

Query	Start	– End	Observed	Mr (expt)	Mr (calc)	ppm	M	Score	Expect	Rank	U	Peptide
752	293	– 304	615.3385	1228.6624	1228.6663	-3.20	0	71	0.00014	1	U	K.ATLDAVVDAVQK.D
753	293	– 304	615.3385	1228.6624	1228.6663	-3.17	0	79	2.1e-05	1	U	K.ATLDAVVDAVQK.D
754	293	– 304	410.5615	1228.6626	1228.6663	-3.04	0	77	3.8e-05	1	U	K.ATLDAVVDAVQK.D
755	293	– 304	615.3387	1228.6629	1228.6663	-2.79	0	97	3.7e-07	1	U	K.ATLDAVVDAVQK.D
756	293	– 304	615.3388	1228.6630	1228.6663	-2.66	0	92	1.1e-06	1	U	K.ATLDAVVDAVQK.D
757	293	– 304	615.3389	1228.6633	1228.6663	-2.45	0	74	8.4e-05	1	U	K.ATLDAVVDAVQK.D
758	293	– 304	615.3389	1228.6633	1228.6663	-2.42	0	87	3.8e-06	1	U	K.ATLDAVVDAVQK.D
759	293	– 304	615.3390	1228.6635	1228.6663	-2.27	0	74	7.8e-05	1	U	K.ATLDAVVDAVQK.D
760	293	– 304	615.3392	1228.6639	1228.6663	-1.98	0	84	6.8e-06	1	U	K.ATLDAVVDAVQK.D
761	293	– 304	615.3393	1228.6641	1228.6663	-1.80	0	86	5.2e-06	1	U	K.ATLDAVVDAVQK.D
762	293	– 304	615.3395	1228.6645	1228.6663	-1.49	0	88	3e-06	1	U	K.ATLDAVVDAVQK.D
763	293	– 304	615.3395	1228.6645	1228.6663	-1.46	0	82	1.1e-05	1	U	K.ATLDAVVDAVQK.D
764	293	– 304	615.3398	1228.6650	1228.6663	-1.10	0	89	2.4e-06	1	U	K.ATLDAVVDAVQK.D
765	293	– 304	615.3408	1228.6671	1228.6663	0.61	0	93	8.8e-07	1	U	K.ATLDAVVDAVQK.D
766	293	– 304	615.3410	1228.6673	1228.6663	0.84	0	87	3.7e-06	1	U	K.ATLDAVVDAVQK.D
767	293	– 304	615.3411	1228.6677	1228.6663	1.16	0	68	0.00031	1	U	K.ATLDAVVDAVQK.D
858	308	– 321	657.8815	1313.7484	1313.7555	-5.37	0	75	3.3e-05	1	U	K.IVIIGGGDTATVAK.K
859	308	– 321	657.8821	1313.7497	1313.7555	-4.38	0	106	2.6e-08	1	U	K.IVIIGGGDTATVAK.K
860	308	– 321	657.8822	1313.7498	1313.7555	-4.30	0	104	4.8e-08	1	U	K.IVIIGGGDTATVAK.K
861	308	– 321	657.8826	1313.7506	1313.7555	-3.72	0	105	3.2e-08	1	U	K.IVIIGGGDTATVAK.K
862	308	– 321	657.8827	1313.7508	1313.7555	-3.54	0	104	4.8e-08	1	U	K.IVIIGGGDTATVAK.K
863	308	– 321	657.8829	1313.7513	1313.7555	-3.16	0	80	1.1e-05	1	U	K.IVIIGGGDTATVAK.K
864	308	– 321	657.8831	1313.7516	1313.7555	-2.98	0	106	2.8e-08	1	U	K.IVIIGGGDTATVAK.K
865	308	– 321	657.8831	1313.7516	1313.7555	-2.95	0	94	4e-07	1	U	K.IVIIGGGDTATVAK.K
866	308	– 321	657.8833	1313.7520	1313.7555	-2.67	0	96	3e-07	1	U	K.IVIIGGGDTATVAK.K
867	308	– 321	657.8833	1313.7520	1313.7555	-2.66	0	92	7.2e-07	1	U	K.IVIIGGGDTATVAK.K
868	308	– 321	657.8834	1313.7523	1313.7555	-2.40	0	106	2.6e-08	1	U	K.IVIIGGGDTATVAK.K
869	308	– 321	657.8835	1313.7523	1313.7555	-2.38	0	105	3.3e-08	1	U	K.IVIIGGGDTATVAK.K
870	308	– 321	657.8835	1313.7524	1313.7555	-2.31	0	103	5.3e-08	1	U	K.IVIIGGGDTATVAK.K
871	308	– 321	657.8835	1313.7525	1313.7555	-2.29	0	80	1.1e-05	1	U	K.IVIIGGGDTATVAK.K
872	308	– 321	657.8835	1313.7525	1313.7555	-2.25	0	106	2.7e-08	1	U	K.IVIIGGGDTATVAK.K
873	308	– 321	657.8835	1313.7525	1313.7555	-2.25	0	105	3.2e-08	1	U	K.IVIIGGGDTATVAK.K
874	308	– 321	657.8837	1313.7529	1313.7555	-1.93	0	99	1.3e-07	1	U	K.IVIIGGGDTATVAK.K

Query	Start	– End	Observed	Mr (expt)	Mr (calc)	ppm	M	Score	Expect	Rank	U	Peptide
875	308	– 321	657.8840	1313.7533	1313.7555	-1.62	0	115	3.2e-09	1	U	K.IVIIGGGDTATVAK.K
876	308	– 321	657.8841	1313.7537	1313.7555	-1.38	0	95	3.6e-07	1	U	K.IVIIGGGDTATVAK.K
877	308	– 321	657.8841	1313.7537	1313.7555	-1.33	0	98	1.5e-07	1	U	K.IVIIGGGDTATVAK.K
878	308	– 321	657.8842	1313.7538	1313.7555	-1.26	0	100	9.8e-08	1	U	K.IVIIGGGDTATVAK.K
879	308	– 321	657.8844	1313.7542	1313.7555	-0.94	0	94	3.8e-07	1	U	K.IVIIGGGDTATVAK.K
880	308	– 321	657.8845	1313.7545	1313.7555	-0.74	0	101	8.4e-08	1	U	K.IVIIGGGDTATVAK.K
881	308	– 321	657.8845	1313.7545	1313.7555	-0.71	0	92	6e-07	1	U	K.IVIIGGGDTATVAK.K
882	308	– 321	657.8847	1313.7548	1313.7555	-0.51	0	105	3.2e-08	1	U	K.IVIIGGGDTATVAK.K
883	308	– 321	657.8847	1313.7548	1313.7555	-0.48	0	94	3.8e-07	1	U	K.IVIIGGGDTATVAK.K
884	308	– 321	657.8851	1313.7557	1313.7555	0.14	0	104	4.5e-08	1	U	K.IVIIGGGDTATVAK.K
885	308	– 321	657.8858	1313.7570	1313.7555	1.16	0	104	4.5e-08	1	U	K.IVIIGGGDTATVAK.K
121	322	– 328	419.7185	837.4223	837.4232	-1.04	1	45	0.05	1	U	K.KYGVEDK.L
1261	329	– 346	877.9631	1753.9116	1753.9210	-5.38	0	107	3e-08	1	U	K.LSHVSTGGGASLELLEGK.E
1263	329	– 346	877.9638	1753.9131	1753.9210	-4.50	0	127	2.7e-10	1	U	K.LSHVSTGGGASLELLEGK.E
1264	329	– 346	585.6458	1753.9157	1753.9210	-3.06	0	72	7.9e-05	1	U	K.LSHVSTGGGASLELLEGK.E
1265	329	– 346	877.9652	1753.9158	1753.9210	-2.96	0	121	1.2e-09	1	U	K.LSHVSTGGGASLELLEGK.E
1266	329	– 346	877.9653	1753.9160	1753.9210	-2.87	0	95	3.9e-07	1	U	K.LSHVSTGGGASLELLEGK.E
1267	329	– 346	585.6461	1753.9165	1753.9210	-2.58	0	63	0.00063	1	U	K.LSHVSTGGGASLELLEGK.E
1268	329	– 346	877.9658	1753.9170	1753.9210	-2.29	0	116	3.7e-09	1	U	K.LSHVSTGGGASLELLEGK.E
1270	329	– 346	585.6463	1753.9172	1753.9210	-2.20	0	60	0.0013	1	U	K.LSHVSTGGGASLELLEGK.E
1271	329	– 346	585.6464	1753.9173	1753.9210	-2.13	0	51	0.011	1	U	K.LSHVSTGGGASLELLEGK.E
1272	329	– 346	877.9659	1753.9173	1753.9210	-2.13	0	119	1.6e-09	1	U	K.LSHVSTGGGASLELLEGK.E
1273	329	– 346	585.6467	1753.9182	1753.9210	-1.64	0	55	0.0046	1	U	K.LSHVSTGGGASLELLEGK.E
1274	329	– 346	585.6467	1753.9183	1753.9210	-1.55	0	68	0.00024	1	U	K.LSHVSTGGGASLELLEGK.E
1275	329	– 346	585.6470	1753.9192	1753.9210	-1.05	0	57	0.003	1	U	K.LSHVSTGGGASLELLEGK.E
1276	329	– 346	877.9674	1753.9203	1753.9210	-0.42	0	119	1.6e-09	1	U	K.LSHVSTGGGASLELLEGK.E
1277	329	– 346	877.9678	1753.9210	1753.9210	0.0040	0	119	1.7e-09	1	U	K.LSHVSTGGGASLELLEGK.E
1278	329	– 346	877.9689	1753.9232	1753.9210	1.26	0	119	1.6e-09	1	U	K.LSHVSTGGGASLELLEGK.E
533	347	– 357	551.3091	1100.6036	1100.6077	-3.76	0	69	0.00021	1	U	K.ELPGVTALSSK.-
538	347	– 357	551.3098	1100.6051	1100.6077	-2.36	0	53	0.0093	1	U	K.ELPGVTALSSK.-

Spot no. 2

Ions score is -10*Log(P), where P is the probability that the observed match is a random event. Individual ions scores > 44 indicate identity or extensive homology (p<0.05). Protein scores are derived from ions scores as a non-probabilistic basis for ranking protein hits.

LL-diaminopimelate aminotransferase, chloroplastic [*Aegilops tauschii*]

Query	Start – End	Observed	Mr(expt)	Mr(calc)	ppm	M	Score	Expect	Rank	U	Peptide
1385	80 – 99	680.6466	2038.9180	2038.9232	-2.55	0	70	8.9e-05	1		R.AHALSTVDGYSGYGAEQGEK.K
1386	80 – 99	1020.4669	2038.9192	2038.9232	-1.94	0	150	7.6e-13	1		R.AHALSTVDGYSGYGAEQGEK.K
1639	143 – 172	1120.2095	3357.6067	3357.6075	-0.25	0	120	6.9e-10	1		K.IAVQDPSYPAYVDSSVIMGQTDLYQQDVQK.Y
1640	143 – 172	1125.5408	3373.6006	3373.6024	-0.55	0	143	3.1e-12	1		K.IAVQDPSYPAYVDSSVIMGQTDLYQQDVQK.Y + Oxidation (M)
421	173 – 180	523.2398	1044.4650	1044.4698	-4.62	0	58	0.0012	1		K.YGNIEYMR.C
451	173 – 180	531.2380	1060.4615	1060.4648	-3.10	0	45	0.02	1		K.YGNIEYMR.C + Oxidation (M)
1077	181 – 193	749.3343	1496.6541	1496.6606	-4.35	0	75	3.4e-05	1		R.CSPENGFFPDLSK.V
1383	197 – 215	680.3247	2037.9524	2037.9578	-2.65	0	86	2.7e-06	1		R.TDIIFFCSPNNPTGAAASR.D
1384	197 – 215	1019.9847	2037.9548	2037.9578	-1.46	0	124	4.2e-10	1		R.TDIIFFCSPNNPTGAAASR.D
680	258 – 268	591.3045	1180.5945	1180.5975	-2.58	0	78	2.6e-05	1		K.EVALETASFSK.Y
193	277 – 284	450.2700	898.5255	898.5276	-2.38	0	46	0.029	1		R.LGWTVVPK.E
866	285 – 296	656.8464	1311.6782	1311.6823	-3.12	0	70	0.00015	1		K.ELLFSDGHPVAK.D
1596	301 – 327	908.7809	2723.3210	2723.3259	-1.79	0	85	3e-06	1		R.IVCTSFNGASTISQAGGLGCLSPEGLK.A
648	328 – 337	579.2882	1156.5619	1156.5587	2.82	0	56	0.0035	1		K.AMQDVVGFYK.E
663	328 – 337	587.2854	1172.5562	1172.5536	2.25	0	62	0.00069	1		K.AMQDVVGFYK.E + Oxidation (M)
1335	383 – 402	953.4748	1904.9350	1904.9381	-1.62	0	103	7.6e-08	1		K.ANVVTTPGSGFGPGGEGFVR.V

putative acyl transferase 4 [*Triticum aestivum*]

Query	Start – End	Observed	Mr(expt)	Mr(calc)	ppm	M	Score	Expect	Rank	U	Peptide
1577	11 – 34	863.7805	2588.3196	2588.3235	-1.50	0	59	0.0013	1	U	R.ISEGAVKPASETPDHTLPLAWVDR.Y
697	40 – 49	594.8121	1187.6097	1187.6121	-2.05	0	67	0.00041	1	U	R.GLVESMHIFR.S
719	40 – 49	602.8077	1203.6008	1203.6070	-5.13	0	49	0.022	1	U	R.GLVESMHIFR.S + Oxidation (M)
397	50 – 60	514.2781	1026.5417	1026.5458	-3.96	0	59	0.0017	1	U	R.SGADAAPAVIR.E
769	148 – 158	622.7969	1243.5792	1243.5842	-4.01	0	78	1.9e-05	1	U	K.FTCGGFVMGLR.F
795	148 – 158	630.7960	1259.5774	1259.5791	-1.34	0	81	8.8e-06	1	U	K.FTCGGFVMGLR.F + Oxidation (M)
1199	159 – 174	555.5987	1663.7742	1663.7777	-2.06	0	47	0.027	1	U	R.FNHASADGMGAAQFIK.A

Query	Start	– End	Observed	Mr(expt)	Mr(calc)	ppm	M	Score	Expect	Rank	U	Peptide
1200	159	– 174	832.8952	1663.7758	1663.7777	-1.10	0	124	5.6e-10	1	U	R.FNHASADGMGAAQFIK.A
1210	159	– 174	560.9308	1679.7707	1679.7726	-1.11	0	53	0.0056	1	U	R.FNHASADGMGAAQFIK.A + Oxidation (M)
1039	182	– 194	740.4005	1478.7864	1478.7882	-1.16	0	45	0.039	1	U	R.GLPEPSVKPVWDR.E
771	241	– 251	622.8021	1243.5897	1243.5907	-0.79	0	86	3.7e-06	1	U	K.FCSGFDVLTAK.L
661	259	– 269	585.8200	1169.6255	1169.6292	-3.16	0	46	0.033	1	U	R.ALNLEPDATVK.L
342	270	– 277	500.2561	998.4976	998.5008	-3.13	0	51	0.009	1	U	K.LCFFASVR.H
1113	282	– 295	772.3923	1542.7701	1542.7718	-1.13	0	99	2.1e-07	1	U	K.LDAGYYGNSIFPVK.M
762	322	– 331	617.3014	1232.5882	1232.5900	-1.44	0	75	5.6e-05	1	U	R.MAVEFFQFAK.E + Oxidation (M)
249	388	– 396	470.2645	938.5145	938.5185	-4.32	0	49	0.016	1	U	K.APLPLDGTR.M
214	397	– 404	455.2301	908.4456	908.4460	-0.36	0	52	0.012	1	U	R.MLASCVTK.E

HSP70 [*Triticum aestivum*]

Query	Start	– End	Observed	Mr(expt)	Mr(calc)	ppm	M	Score	Expect	Rank	U	Peptide
747	28	– 38	614.8156	1227.6166	1227.6207	-3.40	0	78	2.6e-05	1		R.VEIIANDQGNR.T
1071	39	– 51	744.3575	1486.7005	1486.6940	4.35	0	50	0.015	2		R.TTPSYVAFTDTER.L
1181	59	– 73	825.4004	1648.7862	1648.7879	-1.05	0	120	1.4e-09	1		K.NQVAMNPTNTVFDAK.R
1202	59	– 73	833.3956	1664.7766	1664.7828	-3.73	0	128	2e-10	1		K.NQVAMNPTNTVFDAK.R + Oxidation (M)
766	80	– 90	620.7786	1239.5427	1239.5442	-1.19	0	43	0.039	1	U	R.FSDPSVQSDMK.L
786	80	– 90	628.7752	1255.5358	1255.5391	-2.59	0	70	5.6e-05	1	U	R.FSDPSVQSDMK.L + Oxidation (M)
819	132	– 143	639.3382	1276.6619	1276.6663	-3.45	0	70	0.00018	1	U	R.EIAEAFLGNSVK.N
1211	144	– 158	840.9189	1679.8232	1679.8267	-2.09	0	62	0.0011	1		K.NAVVTVPAYFNDSQR.Q
660	163	– 174	585.3338	1168.6530	1168.6564	-2.89	0	104	4.3e-08	1	U	K.DAGAIAGLNVLR.I
1192	175	– 190	553.9688	1658.8847	1658.8879	-1.93	0	67	0.00025	1		R.IINEPTAAAIAYGLDK.K
1193	175	– 190	830.4500	1658.8854	1658.8879	-1.49	0	128	1.9e-10	1		R.IINEPTAAAIAYGLDK.K
1207	226	– 241	838.3670	1674.7195	1674.7234	-2.33	0	99	6.6e-08	1		K.ATAGDTHLGGEDFDNR.M
1208	226	– 241	559.2471	1674.7196	1674.7234	-2.28	0	87	1.1e-06	1		K.ATAGDTHLGGEDFDNR.M
986	334	– 347	476.2590	1425.7553	1425.7576	-1.64	0	45	0.04	2	U	K.STVHDVVLVGGSTR.I

Spot no. 3

Ions score is -10*Log(P), where P is the probability that the observed match is a random event. Individual ions scores > 44 indicate identity or extensive homology (p<0.05). Protein scores are derived from ions scores as a non-probabilistic basis for ranking protein hits.

serpin 1 [*Triticum aestivum*]

Query	Start – End	Observed	Mr(expt)	Mr(calc)	ppm	M	Score	Expect	Rank	U	Peptide
197	11 – 18	463.2633	924.5120	924.5141	-2.24	0	49	0.012	1		R.LSIAHQTR.F
320	23 – 32	509.2806	1016.5466	1016.5502	-3.51	0	48	0.035	1		R.LASTISSNPK.S
321	23 – 32	509.2810	1016.5473	1016.5502	-2.81	0	60	0.0022	1		R.LASTISSNPK.S
960	125 – 137	772.8812	1543.7479	1543.7518	-2.57	1	114	5.9e-09	1		K.YKAETQSVDFQTK.A
610	127 – 137	627.3013	1252.5881	1252.5936	-4.31	0	88	2.4e-06	1		K.AETQSVDFQTK.A
612	127 – 137	627.3050	1252.5955	1252.5936	1.53	0	59	0.002	1		K.AETQSVDFQTK.A
614	127 – 137	627.3072	1252.5999	1252.5936	5.07	0	48	0.028	1		K.AETQSVDFQTK.A
615	127 – 137	627.3080	1252.6015	1252.5936	6.32	0	58	0.0027	1		K.AETQSVDFQTK.A
616	127 – 137	627.3108	1252.6070	1252.5936	10.8	0	64	0.00065	1		K.AETQSVDFQTK.A
981	138 – 151	781.3921	1560.7696	1560.7784	-5.62	0	51	0.015	1		K.AAEVTTQVNSWVEK.V
982	138 – 151	781.3928	1560.7710	1560.7784	-4.71	0	55	0.0054	1		K.AAEVTTQVNSWVEK.V
983	138 – 151	781.3938	1560.7731	1560.7784	-3.40	0	53	0.0086	1		K.AAEVTTQVNSWVEK.V
985	138 – 151	781.3943	1560.7740	1560.7784	-2.78	0	52	0.01	1		K.AAEVTTQVNSWVEK.V
986	138 – 151	781.3943	1560.7741	1560.7784	-2.74	0	111	1.3e-08	1		K.AAEVTTQVNSWVEK.V
987	138 – 151	781.3945	1560.7744	1560.7784	-2.57	0	58	0.0031	1		K.AAEVTTQVNSWVEK.V
988	138 – 151	781.3947	1560.7748	1560.7784	-2.29	0	51	0.016	1		K.AAEVTTQVNSWVEK.V
989	138 – 151	781.3947	1560.7748	1560.7784	-2.28	0	51	0.014	1		K.AAEVTTQVNSWVEK.V
990	138 – 151	781.3948	1560.7750	1560.7784	-2.16	0	52	0.011	1		K.AAEVTTQVNSWVEK.V
991	138 – 151	781.3951	1560.7757	1560.7784	-1.74	0	48	0.03	1		K.AAEVTTQVNSWVEK.V
992	138 – 151	781.3954	1560.7762	1560.7784	-1.42	0	51	0.013	1		K.AAEVTTQVNSWVEK.V
993	138 – 151	781.3954	1560.7762	1560.7784	-1.39	0	51	0.015	1		K.AAEVTTQVNSWVEK.V
994	138 – 151	781.3956	1560.7766	1560.7784	-1.16	0	53	0.01	1		K.AAEVTTQVNSWVEK.V
995	138 – 151	781.3957	1560.7768	1560.7784	-0.98	0	52	0.011	1		K.AAEVTTQVNSWVEK.V
996	138 – 151	781.3958	1560.7770	1560.7784	-0.89	0	52	0.011	1		K.AAEVTTQVNSWVEK.V
997	138 – 151	781.3960	1560.7774	1560.7784	-0.61	0	53	0.0086	1		K.AAEVTTQVNSWVEK.V
998	138 – 151	781.3960	1560.7775	1560.7784	-0.58	0	51	0.014	1		K.AAEVTTQVNSWVEK.V
999	138 – 151	781.3964	1560.7782	1560.7784	-0.085	0	56	0.0047	1		K.AAEVTTQVNSWVEK.V
1000	138 – 151	781.3966	1560.7786	1560.7784	0.13	0	55	0.0057	1		K.AAEVTTQVNSWVEK.V
1001	138 – 151	781.3970	1560.7794	1560.7784	0.63	0	47	0.033	1		K.AAEVTTQVNSWVEK.V
1002	138 – 151	781.3975	1560.7804	1560.7784	1.29	0	50	0.017	1		K.AAEVTTQVNSWVEK.V
1003	138 – 151	781.3975	1560.7804	1560.7784	1.32	0	58	0.0029	1		K.AAEVTTQVNSWVEK.V

Query	Start – End	Observed	Mr(expt)	Mr(calc)	ppm	M	Score	Expect	Rank	U	Peptide
1004	138 – 151	781.4010	1560.7874	1560.7784	5.78	0	51	0.015	1		K.AAEVTTQVNSWVEK.V
745	159 – 171	673.3508	1344.6870	1344.6885	-1.09	0	55	0.0052	1	U	K.NILPSGSVDNTTK.L
471	172 – 181	576.3430	1150.6715	1150.6750	-3.07	0	80	1.3e-05	1		K.LVLANALYFK.G
473	172 – 181	576.3441	1150.6736	1150.6750	-1.21	0	61	0.0012	1		K.LVLANALYFK.G
475	172 – 181	576.3446	1150.6747	1150.6750	-0.26	0	48	0.011	1		K.LVLANALYFK.G
521	262 – 271	588.7963	1175.5781	1175.5822	-3.54	0	80	1.9e-05	1		K.LSAEPDFLER.H
522	262 – 271	588.7973	1175.5800	1175.5822	-1.91	0	60	0.0021	1		K.LSAEPDFLER.H
668	290 – 301	646.8567	1291.6989	1291.7023	-2.65	0	71	0.00013	1		K.ISFGIEASDLLK.C
669	290 – 301	646.8568	1291.6990	1291.7023	-2.62	0	89	1.8e-06	1		K.ISFGIEASDLLK.C
670	290 – 301	646.8569	1291.6992	1291.7023	-2.40	0	74	5.5e-05	1		K.ISFGIEASDLLK.C
671	290 – 301	646.8571	1291.6996	1291.7023	-2.09	0	78	2.3e-05	1		K.ISFGIEASDLLK.C
672	290 – 301	646.8575	1291.7004	1291.7023	-1.53	0	80	1.6e-05	1		K.ISFGIEASDLLK.C
673	290 – 301	646.8576	1291.7007	1291.7023	-1.28	0	84	5.6e-06	1		K.ISFGIEASDLLK.C
674	290 – 301	646.8578	1291.7010	1291.7023	-1.02	0	84	5.9e-06	1		K.ISFGIEASDLLK.C
675	290 – 301	646.8579	1291.7013	1291.7023	-0.84	0	76	3.7e-05	1		K.ISFGIEASDLLK.C
676	290 – 301	646.8579	1291.7013	1291.7023	-0.82	0	84	5.8e-06	1		K.ISFGIEASDLLK.C
677	290 – 301	646.8581	1291.7016	1291.7023	-0.54	0	55	0.0044	1		K.ISFGIEASDLLK.C
678	290 – 301	646.8581	1291.7017	1291.7023	-0.49	0	67	0.00031	1		K.ISFGIEASDLLK.C
679	290 – 301	646.8582	1291.7018	1291.7023	-0.40	0	72	9.1e-05	1		K.ISFGIEASDLLK.C
680	290 – 301	646.8582	1291.7019	1291.7023	-0.36	0	76	3.9e-05	1		K.ISFGIEASDLLK.C
681	290 – 301	646.8583	1291.7021	1291.7023	-0.19	0	60	0.0015	1		K.ISFGIEASDLLK.C
682	290 – 301	646.8591	1291.7036	1291.7023	0.98	0	71	0.00011	1		K.ISFGIEASDLLK.C
1544	329 – 354	680.8418	2719.3382	2719.3453	-2.63	0	94	4.3e-07	1		R.VSSVFHQAFVEVNEQGTEAAASTAIK.M
1545	329 – 354	907.4538	2719.3396	2719.3453	-2.11	0	155	3.1e-13	1		R.VSSVFHQAFVEVNEQGTEAAASTAIK.M
1546	329 – 354	680.8422	2719.3398	2719.3453	-2.04	0	79	1.2e-05	1		R.VSSVFHQAFVEVNEQGTEAAASTAIK.M
1547	329 – 354	907.4540	2719.3401	2719.3453	-1.94	0	147	1.9e-12	1		R.VSSVFHQAFVEVNEQGTEAAASTAIK.M
1548	329 – 354	907.4542	2719.3408	2719.3453	-1.67	0	155	3.2e-13	1		R.VSSVFHQAFVEVNEQGTEAAASTAIK.M
1549	329 – 354	1360.6784	2719.3422	2719.3453	-1.13	0	169	1.2e-14	1		R.VSSVFHQAFVEVNEQGTEAAASTAIK.M
1550	329 – 354	907.4549	2719.3428	2719.3453	-0.92	0	160	1.1e-13	1		R.VSSVFHQAFVEVNEQGTEAAASTAIK.M

Spot no. 4

Ions score is -10*Log(P), where P is the probability that the observed match is a random event. Individual ions scores > 44 indicate identity or extensive homology ($p<0.05$). Protein scores are derived from ions scores as a non-probabilistic basis for ranking protein hits.

predicted protein [*Hordeum vulgare subsp. vulgare*]

Query	Start – End	Observed	Mr(expt)	Mr(calc)	ppm	M	Score	Expect	Rank	U	Peptide
848	36 – 50	667.8791	1333.7436	1333.7466	-2.27	0	115	3.6e-09	1	U	K.APGGAPANVAIAVAR.L
202	51 – 60	438.7500	875.4853	875.4865	-1.33	0	74	8.6e-05	1	U	R.LGGGAAFVGK.L
241	61 – 68	454.7086	907.4026	907.4036	-1.03	0	70	0.00011	1	U	K.LGDDEFGR.M
1052	76 – 91	768.3565	1534.6985	1534.7012	-1.79	0	104	4.6e-08	1	U	R.DNGVDAGGVVFDSGAR.T
1054	76 – 91	768.3569	1534.6993	1534.7012	-1.24	0	95	3.9e-07	1	U	R.DNGVDAGGVVFDSGAR.T
1055	76 – 91	768.3574	1534.7003	1534.7012	-0.58	0	49	0.013	1	U	R.DNGVDAGGVVFDSGAR.T
350	92 – 100	496.2992	990.5838	990.5862	-2.47	0	47	0.019	1		R.TALAFVTLR.A
1227	132 – 148	945.9780	1889.9415	1889.9458	-2.26	0	80	1.5e-05	1	U	R.AAVFHYGSISLIAEPCR.T
940	160 – 172	709.8669	1417.7192	1417.7201	-0.66	0	51	0.016	1		K.EAGALLSYDPNLR.E
806	173 – 183	650.8292	1299.6438	1299.6459	-1.57	0	49	0.018	1	U	R.EALWPSLEEAR.T
917	186 – 197	700.8913	1399.7679	1399.7711	-2.25	0	102	8.4e-08	1		K.ILSIWDQADIVK.V
1270	253 – 271	974.5080	1947.0014	1947.0062	-2.46	0	169	1.8e-14	1		K.VQQVDTTGAGDAFIGSLLR.K
851	272 – 283	447.9171	1340.7296	1340.7300	-0.28	1	62	0.00076	1		R.KIVQDPSALQDK.K
852	272 – 283	671.3722	1340.7299	1340.7300	-0.043	1	93	6e-07	1		R.KIVQDPSALQDK.K
687	273 – 283	607.3237	1212.6329	1212.6350	-1.73	0	72	9.6e-05	1		K.IVQDPSALQDK.K
723	292 – 303	612.8045	1223.5944	1223.5968	-1.98	0	86	3.6e-06	1		K.FANACGAITATK.K
249	304 – 312	455.7888	909.5631	909.5647	-1.80	1	45	0.012	1		K.KGAIPSLPK.E

putative NADP-dependent oxidoreductase P1 [*Triticum urartu*]

Query	Start – End	Observed	Mr(expt)	Mr(calc)	ppm	M	Score	Expect	Rank	U	Peptide
1305	17 – 35	1049.4923	2096.9700	2096.9725	-1.16	0	69	0.00013	1	U	R.YVTGFPSEDDMELVPATAR.L
924	49 – 59	707.3428	1412.6711	1412.6758	-3.32	0	69	0.00019	1		K.NLYLSCDPYLR.S
926	49 – 59	707.3447	1412.6748	1412.6758	-0.71	0	55	0.0042	1		K.NLYLSCDPYLR.S
927	49 – 59	707.3448	1412.6751	1412.6758	-0.53	0	52	0.0097	1		K.NLYLSCDPYLR.S
931	49 – 59	707.3459	1412.6772	1412.6758	1.00	0	47	0.028	1		K.NLYLSCDPYLR.S
932	49 – 59	707.3460	1412.6773	1412.6758	1.09	0	52	0.0095	1		K.NLYLSCDPYLR.S
934	49 – 59	707.3467	1412.6788	1412.6758	2.11	0	56	0.0036	1		K.NLYLSCDPYLR.S
1495	62 – 87	915.1094	2742.3063	2742.3171	-3.95	0	108	1.4e-08	1	U	R.MSGNDEPSHVPDFVQGEVLTTLGVSK.V
1496	62 – 87	1372.1627	2742.3108	2742.3171	-2.28	0	126	2.5e-10	1	U	R.MSGNDEPSHVPDFVQGEVLTTLGVSK.V
1497	62 – 87	915.1110	2742.3112	2742.3171	-2.15	0	114	3.7e-09	1	U	R.MSGNDEPSHVPDFVQGEVLTTLGVSK.V

Query	Start – End		Observed	Mr(expt)	Mr(calc)	ppm	M	Score	Expect	Rank	U	Peptide
1498	62	– 87	915.1138	2742.3196	2742.3171	0.90	0	124	3.5e-10	1	U	R.MSGNDEPSHVPDFVQGEVLTTLGVSK.V
1500	62	– 87	920.4414	2758.3025	2758.3120	-3.46	0	118	1.6e-09	1	U	R.MSGNDEPSHVPDFVQGEVLTTLGVSK.V + Oxidation (M)
1501	62	– 87	920.4427	2758.3064	2758.3120	-2.04	0	135	3.2e-11	1	U	R.MSGNDEPSHVPDFVQGEVLTTLGVSK.V + Oxidation (M)
1502	62	– 87	1380.1611	2758.3076	2758.3120	-1.58	0	120	9.6e-10	1	U	R.MSGNDEPSHVPDFVQGEVLTTLGVSK.V + Oxidation (M)
1503	62	– 87	920.4449	2758.3129	2758.3120	0.33	0	146	2.5e-12	1	U	R.MSGNDEPSHVPDFVQGEVLTTLGVSK.V + Oxidation (M)
1504	62	– 87	1380.1693	2758.3240	2758.3120	4.36	0	101	7.1e-08	1	U	R.MSGNDEPSHVPDFVQGEVLTTLGVSK.V + Oxidation (M)
1027	179	– 193	764.8445	1527.6745	1527.6875	-8.52	0	54	0.003	1		K.ISGCYVVGSAGSDEK.V
1028	179	– 193	764.8488	1527.6831	1527.6875	-2.91	0	102	6e-08	1		K.ISGCYVVGSAGSDEK.V
1029	179	– 193	510.2352	1527.6837	1527.6875	-2.54	0	87	1.8e-06	1		K.ISGCYVVGSAGSDEK.V
1030	179	– 193	764.8495	1527.6844	1527.6875	-2.07	0	102	5.2e-08	1		K.ISGCYVVGSAGSDEK.V
1031	179	– 193	764.8495	1527.6844	1527.6875	-2.06	0	111	7.3e-09	1		K.ISGCYVVGSAGSDEK.V
1032	179	– 193	764.8511	1527.6876	1527.6875	0.037	0	116	2.1e-09	1		K.ISGCYVVGSAGSDEK.V
1033	179	– 193	764.8512	1527.6878	1527.6875	0.16	0	50	0.0087	1		K.ISGCYVVGSAGSDEK.V
1034	179	– 193	764.8513	1527.6880	1527.6875	0.33	0	50	0.0086	1		K.ISGCYVVGSAGSDEK.V
1036	179	– 193	764.8520	1527.6894	1527.6875	1.24	0	110	9.5e-09	1		K.ISGCYVVGSAGSDEK.V
1037	179	– 193	764.8520	1527.6895	1527.6875	1.29	0	102	6.2e-08	1		K.ISGCYVVGSAGSDEK.V
1038	179	– 193	764.8522	1527.6899	1527.6875	1.56	0	102	6.1e-08	1		K.ISGCYVVGSAGSDEK.V
1039	179	– 193	764.8523	1527.6901	1527.6875	1.69	0	97	1.8e-07	1		K.ISGCYVVGSAGSDEK.V
1040	179	– 193	764.8524	1527.6903	1527.6875	1.80	0	98	1.4e-07	1		K.ISGCYVVGSAGSDEK.V
1041	179	– 193	764.8524	1527.6903	1527.6875	1.80	0	93	4.5e-07	1		K.ISGCYVVGSAGSDEK.V
1042	179	– 193	764.8525	1527.6905	1527.6875	1.94	0	82	6.6e-06	1		K.ISGCYVVGSAGSDEK.V
1043	179	– 193	764.8529	1527.6912	1527.6875	2.43	0	93	5.4e-07	1		K.ISGCYVVGSAGSDEK.V
1044	179	– 193	764.8543	1527.6941	1527.6875	4.28	0	92	5.9e-07	1		K.ISGCYVVGSAGSDEK.V
1045	179	– 193	764.8546	1527.6946	1527.6875	4.66	0	53	0.0051	1		K.ISGCYVVGSAGSDEK.V
701	201	– 210	612.2703	1222.5260	1222.5295	-2.85	0	79	8.8e-06	1		K.FGFDDAFNYK.K
703	201	– 210	612.2706	1222.5266	1222.5295	-2.39	0	71	5.4e-05	1		K.FGFDDAFNYK.K
704	201	– 210	1223.5343	1222.5270	1222.5295	-2.02	0	44	0.027	1		K.FGFDDAFNYK.K
706	201	– 210	612.2710	1222.5274	1222.5295	-1.74	0	63	0.00032	1		K.FGFDDAFNYK.K
713	201	– 210	612.2718	1222.5291	1222.5295	-0.32	0	53	0.0041	1		K.FGFDDAFNYK.K
584	211	– 220	580.8111	1159.6076	1159.6084	-0.74	1	94	9.3e-07	1	U	K.KEQDLDATLK.R
415	212	– 220	516.7640	1031.5134	1031.5135	-0.059	0	49	0.023	1	U	K.EQDLDATLK.R
630	212	– 221	594.8137	1187.6129	1187.6146	-1.44	1	53	0.0094	1	U	K.EQDLDATLKR.C
1324	252	– 270	1069.5290	2137.0434	2137.0474	-1.83	0	117	2.6e-09	1	U	R.VSVCGLISQYNLEQSEGVR.N

Query	Start – End	Observed	Mr(expt)	Mr(calc)	ppm	M	Score	Expect	Rank	U	Peptide
374	271 – 278	504.7794	1007.5443	1007.5474	-3.09	0	46	0.031	1	U	R.NLFCIITK.R
854	296 – 306	672.8262	1343.6379	1343.6431	-3.83	1	73	9.2e-05	1		R.KFEEEMAGYLK.D
859	296 – 306	672.8284	1343.6423	1343.6431	-0.59	1	69	0.00022	1		R.KFEEEMAGYLK.D
860	296 – 306	672.8288	1343.6431	1343.6431	-0.0067	1	91	1.5e-06	1		R.KFEEEMAGYLK.D
861	296 – 306	672.8299	1343.6453	1343.6431	1.62	1	56	0.0044	1		R.KFEEEMAGYLK.D
862	296 – 306	672.8300	1343.6454	1343.6431	1.69	1	57	0.0042	1		R.KFEEEMAGYLK.D
885	296 – 306	680.8228	1359.6310	1359.6380	-5.14	1	53	0.0064	1		R.KFEEEMAGYLK.D + Oxidation (M)
887	296 – 306	680.8249	1359.6352	1359.6380	-2.09	1	86	3.1e-06	1		R.KFEEEMAGYLK.D + Oxidation (M)
888	296 – 306	454.2192	1359.6356	1359.6380	-1.76	1	54	0.0049	1		R.KFEEEMAGYLK.D + Oxidation (M)
688	297 – 306	608.7799	1215.5453	1215.5481	-2.31	0	85	3.7e-06	1		K.FEEEMAGYLK.D
689	297 – 306	608.7814	1215.5483	1215.5481	0.12	0	53	0.0072	1		K.FEEEMAGYLK.D
726	297 – 306	616.7780	1231.5415	1231.5431	-1.28	0	79	8.7e-06	1		K.FEEEMAGYLK.D + Oxidation (M)

Spot no. 5

Ions score is -10*Log(P), where P is the probability that the observed match is a random event. Individual ions scores > 44 indicate identity or extensive homology (p<0.05). Protein scores are derived from ions scores as a non-probabilistic basis for ranking protein hits.

predicted protein [*Hordeum vulgare subsp. vulgare*]

Query	Start – End	Observed	Mr(expt)	Mr(calc)	ppm	M	Score	Expect	Rank	U	Peptide
1099	109 – 125	918.4879	1834.9612	1834.9652	-2.14	0	61	0.00091	1	U	K.VVLFAVPGAFTPTCTQK.H
1103	141 – 157	920.4399	1838.8652	1838.8655	-0.16	0	95	3.7e-07	1	U	K.GVDTVACVSVNDAFVMR.A
1104	141 – 157	920.4415	1838.8685	1838.8655	1.61	0	131	1e-10	1	U	K.GVDTVACVSVNDAFVMR.A
1116	141 – 157	928.4317	1854.8488	1854.8605	-6.28	0	87	2.4e-06	1	U	K.GVDTVACVSVNDAFVMR.A + Oxidation (M)
1117	141 – 157	928.4334	1854.8523	1854.8605	-4.40	0	87	2.3e-06	1	U	K.GVDTVACVSVNDAFVMR.A + Oxidation (M)
1323	161 – 181	1087.0518	2172.0890	2172.0910	-0.90	0	95	4.5e-07	1	U	K.ESLGVGDEVLLLSDGNGELTR.A
1324	161 – 181	1087.0541	2172.0936	2172.0910	1.22	0	105	3.9e-08	1	U	K.ESLGVGDEVLLLSDGNGELTR.A
1325	161 – 181	1087.0542	2172.0938	2172.0910	1.31	0	138	2.1e-11	1	U	K.ESLGVGDEVLLLSDGNGELTR.A
1326	161 – 181	1087.0626	2172.1106	2172.0910	9.04	0	123	6e-10	1	U	K.ESLGVGDEVLLLSDGNGELTR.A
1118	182 – 199	619.3351	1854.9834	1854.9873	-2.10	0	102	7.5e-08	1	U	R.AMGVELDLSDKPVGLGVR.S
1119	182 – 199	619.3352	1854.9837	1854.9873	-1.95	0	113	6.2e-09	1	U	R.AMGVELDLSDKPVGLGVR.S
1120	182 – 199	928.4997	1854.9849	1854.9873	-1.32	0	114	5.1e-09	1	U	R.AMGVELDLSDKPVGLGVR.S
1130	182 – 199	624.6675	1870.9807	1870.9823	-0.83	0	79	1.8e-05	1	U	R.AMGVELDLSDKPVGLGVR.S + Oxidation (M)

Query	Start – End	Observed	Mr(expt)	Mr(calc)	ppm	M	Score	Expect	Rank	U	Peptide
1131	182 – 199	936.4984	1870.9823	1870.9823	0.0075	0	101	9.6e-08	1	U	R.AMGVELDLSDKPVGLGVR.S + Oxidation (M)
1132	182 – 199	624.6683	1870.9830	1870.9823	0.37	0	53	0.0064	1	U	R.AMGVELDLSDKPVGLGVR.S + Oxidation (M)
680	202 – 213	440.5823	1318.7252	1318.7245	0.53	1	57	0.0025	1		R.RYALLADDGVVK.V
681	202 – 213	660.3708	1318.7270	1318.7245	1.90	1	44	0.049	1		R.RYALLADDGVVK.V
538	203 – 213	582.3185	1162.6224	1162.6234	-0.86	0	74	8e-05	1		R.YALLADDGVVK.V
1309	214 – 233	709.0089	2124.0049	2124.0045	0.19	0	79	1.3e-05	1		K.VLNLEEGGAFTNSSAEDMLK.A
1310	214 – 233	1063.0102	2124.0058	2124.0045	0.65	0	178	1.9e-15	1		K.VLNLEEGGAFTNSSAEDMLK.A
1311	214 – 233	1063.0110	2124.0074	2124.0045	1.40	0	178	1.8e-15	1		K.VLNLEEGGAFTNSSAEDMLK.A
1314	214 – 233	1071.0039	2139.9932	2139.9994	-2.87	0	133	5.4e-11	1		K.VLNLEEGGAFTNSSAEDMLK.A + Oxidation (M)
1315	214 – 233	1071.0062	2139.9978	2139.9994	-0.72	0	167	2.3e-14	1		K.VLNLEEGGAFTNSSAEDMLK.A + Oxidation (M)
1316	214 – 233	714.3401	2139.9984	2139.9994	-0.47	0	76	2.8e-05	1		K.VLNLEEGGAFTNSSAEDMLK.A + Oxidation (M)

dimeric alpha-amylase inhibitor [*Triticum dicoccoides*]

Query	Start – End	Observed	Mr(expt)	Mr(calc)	ppm	M	Score	Expect	Rank	U	Peptide
965	26 – 39	785.9044	1569.7943	1569.7933	0.60	0	82	8.8e-06	1		K.LQCNGSQVPEAVLR.D
1105	40 – 53	920.8740	1839.7334	1839.7338	-0.26	0	123	9.7e-11	1		R.DCCQQLADISEWCR.C
946	54 – 66	777.8343	1553.6540	1553.6564	-1.57	0	85	1.7e-06	1		R.CGALYSMLDSMYK.E + Oxidation (M)
947	54 – 66	777.8348	1553.6550	1553.6564	-0.92	0	100	5.5e-08	1		R.CGALYSMLDSMYK.E + Oxidation (M)
1143	67 – 84	944.4230	1886.8315	1886.8330	-0.79	0	141	5.9e-12	1		K.EHGVQEGQAGTGAFPSCR.R
1144	67 – 84	629.9518	1886.8336	1886.8330	0.32	0	46	0.022	1		K.EHGVQEGQAGTGAFPSCR.R
510	90 – 100	567.8116	1133.6086	1133.6114	-2.48	0	86	4.5e-06	1		K.LTAASITAVCK.L
1018	101 – 116	839.4291	1676.8437	1676.8444	-0.39	0	122	9.8e-10	1		K.LPIVIDASGDGAYVCK.G

Spot no. 6

Ions score is -10*Log(P), where P is the probability that the observed match is a random event. Individual ions scores > 43 indicate identity or extensive homology (p<0.05). Protein scores are derived from ions scores as a non-probabilistic basis for ranking protein hits.

0.19 dimeric alpha-amylase inhibitor [*Aegilops tauschii*]

Query	Start – End	Observed	Mr(expt)	Mr(calc)	ppm	M	Score	Expect	Rank	U	Peptide
685	26 – 39	785.9043	1569.7941	1569.7933	0.51	0	84	6.6e-06	1		R.LQCNGSQVPEAVLR.D
816	40 – 53	621.5952	1861.7637	1861.7658	-1.11	0	43	0.014	1		R.DCCQQLAHISEWCR.C

Query	Start – End	Observed	Mr(expt)	Mr(calc)	ppm	M	Score	Expect	Rank	U	Peptide
684	54 – 66	785.8356	1569.6566	1569.6513	3.37	0	61	0.00034	1		R.CGALYSMLDSMYK.X + 2 Oxidation (M)
708	67 – 82	806.3608	1610.7070	1610.7550	-29.8	0	118	1.2e-09	1	U	K.QHGAQEGQAGTGAFPR.C
709	67 – 82	538.2537	1611.7394	1611.7390	0.25	0	52	0.0076	1	U	K.EHGAQEGQAGTGAFPR.C
710	67 – 82	806.8781	1611.7417	1611.7390	1.70	0	122	7.7e-10	1	U	K.EHGAQEGQAGTGAFPR.C
388	90 – 100	581.8147	1161.6149	1161.6176	-2.29	0	81	1.6e-05	1		K.LTAASITAVCR.L
729	101 – 116	832.4229	1662.8311	1662.8287	1.46	0	122	1.1e-09	1		R.LPIVVDASGDGAYVCK.D

Spot no. 7

Ions score is -10*Log(P), where P is the probability that the observed match is a random event. Individual ions scores > 43 indicate identity or extensive homology (p<0.05). Protein scores are derived from ions scores as a non-probabilistic basis for ranking protein hits.

predicted protein [*Hordeum vulgare subsp. vulgare*]

Query	Start – End	Observed	Mr(expt)	Mr(calc)	ppm	M	Score	Expect	Rank	U	Peptide
1263	7 – 25	1130.5167	2259.0188	2259.0273	-3.75	0	121	6.7e-10	1		R.SNVFDPFADLWADPFDTFR.S
1264	7 – 25	1130.5187	2259.0228	2259.0273	-1.98	0	121	6e-10	1		R.SNVFDPFADLWADPFDTFR.S
1140	26 – 45	966.9867	1931.9588	1931.9701	-5.84	0	125	4.2e-10	1	U	R.SIVPAISGGNSETAAFANAR.M
1141	26 – 45	966.9899	1931.9653	1931.9701	-2.49	0	81	1.1e-05	1	U	R.SIVPAISGGNSETAAFANAR.M
1142	26 – 45	966.9908	1931.9671	1931.9701	-1.55	0	81	1.1e-05	1	U	R.SIVPAISGGNSETAAFANAR.M
1143	26 – 45	644.9963	1931.9671	1931.9701	-1.54	0	60	0.0016	1	U	R.SIVPAISGGNSETAAFANAR.M
1144	26 – 45	966.9910	1931.9674	1931.9701	-1.38	0	153	6.5e-13	1	U	R.SIVPAISGGNSETAAFANAR.M
1145	26 – 45	644.9972	1931.9698	1931.9701	-0.14	0	53	0.007	1	U	R.SIVPAISGGNSETAAFANAR.M
1147	26 – 45	966.9931	1931.9716	1931.9701	0.81	0	163	7.5e-14	1	U	R.SIVPAISGGNSETAAFANAR.M
1148	26 – 45	966.9931	1931.9716	1931.9701	0.81	0	156	3.8e-13	1	U	R.SIVPAISGGNSETAAFANAR.M
1149	26 – 45	966.9943	1931.9740	1931.9701	2.04	0	90	1.3e-06	1	U	R.SIVPAISGGNSETAAFANAR.M
1150	26 – 45	966.9969	1931.9792	1931.9701	4.72	0	114	6.3e-09	1	U	R.SIVPAISGGNSETAAFANAR.M
1211	67 – 85	696.0274	2085.0605	2085.0590	0.73	1	72	7.9e-05	1		K.EEVKVEVEDGNVLVVSGER.T
1212	67 – 85	1043.5378	2085.0610	2085.0590	0.99	1	129	1.6e-10	1		K.EEVKVEVEDGNVLVVSGER.T
1213	67 – 85	696.0287	2085.0643	2085.0590	2.57	1	54	0.0048	1		K.EEVKVEVEDGNVLVVSGER.T
893	71 – 85	800.9073	1599.8000	1599.8104	-6.50	0	125	5.2e-10	1		K.VEVEDGNVLVVSGER.T
894	71 – 85	800.9096	1599.8046	1599.8104	-3.65	0	126	3.6e-10	1		K.VEVEDGNVLVVSGER.T
895	71 – 85	800.9099	1599.8052	1599.8104	-3.29	0	115	5.1e-09	1		K.VEVEDGNVLVVSGER.T
896	71 – 85	800.9103	1599.8060	1599.8104	-2.76	0	115	5.1e-09	1		K.VEVEDGNVLVVSGER.T

Query	Start – End	Observed	Mr(expt)	Mr(calc)	ppm	M	Score	Expect	Rank	U	Peptide
897	71 – 85	800.9103	1599.8060	1599.8104	-2.74	0	122	9.2e-10	1		K.VEVEDGNVLVVSGER.T
898	71 – 85	800.9106	1599.8066	1599.8104	-2.36	0	113	7.9e-09	1		K.VEVEDGNVLVVSGER.T
899	71 – 85	800.9111	1599.8075	1599.8104	-1.80	0	115	4.7e-09	1		K.VEVEDGNVLVVSGER.T
900	71 – 85	800.9112	1599.8079	1599.8104	-1.60	0	115	4.6e-09	1		K.VEVEDGNVLVVSGER.T
901	71 – 85	800.9112	1599.8079	1599.8104	-1.59	0	124	6.8e-10	1		K.VEVEDGNVLVVSGER.T
902	71 – 85	534.2767	1599.8081	1599.8104	-1.44	0	49	0.018	1		K.VEVEDGNVLVVSGER.T
903	71 – 85	800.9115	1599.8084	1599.8104	-1.24	0	122	1.1e-09	1		K.VEVEDGNVLVVSGER.T
904	71 – 85	800.9116	1599.8087	1599.8104	-1.10	0	115	4.7e-09	1		K.VEVEDGNVLVVSGER.T
905	71 – 85	800.9116	1599.8087	1599.8104	-1.09	0	130	1.5e-10	1		K.VEVEDGNVLVVSGER.T
906	71 – 85	800.9117	1599.8089	1599.8104	-0.95	0	131	1.3e-10	1		K.VEVEDGNVLVVSGER.T
907	71 – 85	800.9117	1599.8089	1599.8104	-0.92	0	124	6.9e-10	1		K.VEVEDGNVLVVSGER.T
908	71 – 85	800.9118	1599.8091	1599.8104	-0.85	0	124	6.8e-10	1		K.VEVEDGNVLVVSGER.T
909	71 – 85	800.9119	1599.8092	1599.8104	-0.75	0	126	3.8e-10	1		K.VEVEDGNVLVVSGER.T
910	71 – 85	800.9121	1599.8095	1599.8104	-0.55	0	124	7e-10	1		K.VEVEDGNVLVVSGER.T
911	71 – 85	800.9122	1599.8098	1599.8104	-0.37	0	123	8.6e-10	1		K.VEVEDGNVLVVSGER.T
912	71 – 85	800.9123	1599.8101	1599.8104	-0.21	0	120	1.6e-09	1		K.VEVEDGNVLVVSGER.T
913	71 – 85	800.9124	1599.8103	1599.8104	-0.073	0	113	7.7e-09	1		K.VEVEDGNVLVVSGER.T
914	71 – 85	800.9124	1599.8103	1599.8104	-0.061	0	115	4.6e-09	1		K.VEVEDGNVLVVSGER.T
915	71 – 85	800.9126	1599.8106	1599.8104	0.11	0	123	7.5e-10	1		K.VEVEDGNVLVVSGER.T
916	71 – 85	534.2775	1599.8108	1599.8104	0.23	0	66	0.00036	1		K.VEVEDGNVLVVSGER.T
917	71 – 85	800.9127	1599.8109	1599.8104	0.28	0	126	4e-10	1		K.VEVEDGNVLVVSGER.T
918	71 – 85	800.9127	1599.8109	1599.8104	0.28	0	122	1.1e-09	1		K.VEVEDGNVLVVSGER.T
919	71 – 85	800.9127	1599.8109	1599.8104	0.30	0	120	1.6e-09	1		K.VEVEDGNVLVVSGER.T
920	71 – 85	800.9127	1599.8109	1599.8104	0.33	0	126	3.8e-10	1		K.VEVEDGNVLVVSGER.T
921	71 – 85	800.9128	1599.8110	1599.8104	0.36	0	124	6.8e-10	1		K.VEVEDGNVLVVSGER.T
922	71 – 85	800.9128	1599.8110	1599.8104	0.39	0	115	5.1e-09	1		K.VEVEDGNVLVVSGER.T
923	71 – 85	800.9128	1599.8110	1599.8104	0.39	0	115	4.5e-09	1		K.VEVEDGNVLVVSGER.T
924	71 – 85	800.9128	1599.8110	1599.8104	0.39	0	135	5e-11	1		K.VEVEDGNVLVVSGER.T
925	71 – 85	800.9129	1599.8113	1599.8104	0.54	0	115	4.5e-09	1		K.VEVEDGNVLVVSGER.T
926	71 – 85	800.9130	1599.8115	1599.8104	0.65	0	115	4.6e-09	1		K.VEVEDGNVLVVSGER.T
927	71 – 85	800.9132	1599.8119	1599.8104	0.90	0	137	3.2e-11	1		K.VEVEDGNVLVVSGER.T
928	71 – 85	800.9132	1599.8119	1599.8104	0.91	0	120	1.6e-09	1		K.VEVEDGNVLVVSGER.T
929	71 – 85	800.9134	1599.8122	1599.8104	1.14	0	124	6.8e-10	1		K.VEVEDGNVLVVSGER.T

Query	Start – End	Observed	Mr(expt)	Mr(calc)	ppm	M	Score	Expect	Rank	U	Peptide
930	71 – 85	800.9134	1599.8122	1599.8104	1.14	0	120	1.7e-09	1		K.VEVEDGNVLVVSGER.T
931	71 – 85	800.9135	1599.8125	1599.8104	1.30	0	113	7.5e-09	1		K.VEVEDGNVLVVSGER.T
932	71 – 85	800.9139	1599.8132	1599.8104	1.71	0	120	1.7e-09	1		K.VEVEDGNVLVVSGER.T
933	71 – 85	800.9139	1599.8132	1599.8104	1.76	0	112	9.4e-09	1		K.VEVEDGNVLVVSGER.T
934	71 – 85	800.9144	1599.8142	1599.8104	2.34	0	131	1.2e-10	1		K.VEVEDGNVLVVSGER.T
935	71 – 85	800.9158	1599.8170	1599.8104	4.09	0	124	6.8e-10	1		K.VEVEDGNVLVVSGER.T
269	110 – 117	488.2655	974.5165	974.5185	-2.05	1	49	0.033	1		R.FRLPEDAK.V
689	123 – 136	699.3974	1396.7802	1396.7926	-8.89	0	64	0.00039	1		K.AGLENGVLTVTVPK.A
690	123 – 136	699.3980	1396.7814	1396.7926	-7.99	0	69	0.00013	1		K.AGLENGVLTVTVPK.A
691	123 – 136	699.3988	1396.7830	1396.7926	-6.84	0	71	7.8e-05	1		K.AGLENGVLTVTVPK.A
692	123 – 136	699.3992	1396.7839	1396.7926	-6.21	0	68	0.00014	1		K.AGLENGVLTVTVPK.A
693	123 – 136	699.3993	1396.7840	1396.7926	-6.17	0	68	0.00016	1		K.AGLENGVLTVTVPK.A
694	123 – 136	699.3993	1396.7841	1396.7926	-6.08	0	74	4.1e-05	1		K.AGLENGVLTVTVPK.A
695	123 – 136	699.3994	1396.7843	1396.7926	-5.96	0	62	0.00057	1		K.AGLENGVLTVTVPK.A
696	123 – 136	699.3995	1396.7844	1396.7926	-5.86	0	75	2.9e-05	1		K.AGLENGVLTVTVPK.A
697	123 – 136	699.3997	1396.7849	1396.7926	-5.53	0	62	0.00053	1		K.AGLENGVLTVTVPK.A
698	123 – 136	699.3998	1396.7850	1396.7926	-5.40	0	63	0.00042	1		K.AGLENGVLTVTVPK.A
699	123 – 136	699.3999	1396.7852	1396.7926	-5.31	0	57	0.002	1		K.AGLENGVLTVTVPK.A
700	123 – 136	699.3999	1396.7853	1396.7926	-5.21	0	59	0.0011	1		K.AGLENGVLTVTVPK.A
701	123 – 136	699.3999	1396.7853	1396.7926	-5.20	0	66	0.00023	1		K.AGLENGVLTVTVPK.A
702	123 – 136	699.3999	1396.7853	1396.7926	-5.20	0	77	1.8e-05	1		K.AGLENGVLTVTVPK.A
703	123 – 136	699.4000	1396.7854	1396.7926	-5.15	0	68	0.00014	1		K.AGLENGVLTVTVPK.A
704	123 – 136	699.4002	1396.7859	1396.7926	-4.80	0	78	1.6e-05	1		K.AGLENGVLTVTVPK.A
705	123 – 136	699.4004	1396.7862	1396.7926	-4.55	0	73	4.9e-05	1		K.AGLENGVLTVTVPK.A
706	123 – 136	699.4007	1396.7868	1396.7926	-4.15	0	66	0.00023	1		K.AGLENGVLTVTVPK.A
707	123 – 136	699.4010	1396.7874	1396.7926	-3.72	0	57	0.0018	1		K.AGLENGVLTVTVPK.A
708	123 – 136	699.4010	1396.7874	1396.7926	-3.69	0	72	5e-05	1		K.AGLENGVLTVTVPK.A
709	123 – 136	699.4010	1396.7875	1396.7926	-3.66	0	69	0.00012	1		K.AGLENGVLTVTVPK.A
710	123 – 136	699.4011	1396.7876	1396.7926	-3.55	0	69	0.00011	1		K.AGLENGVLTVTVPK.A
711	123 – 136	699.4011	1396.7877	1396.7926	-3.51	0	83	4.4e-06	1		K.AGLENGVLTVTVPK.A
712	123 – 136	699.4012	1396.7877	1396.7926	-3.46	0	59	0.0012	1		K.AGLENGVLTVTVPK.A
713	123 – 136	699.4012	1396.7878	1396.7926	-3.45	0	57	0.0018	1		K.AGLENGVLTVTVPK.A
714	123 – 136	699.4015	1396.7884	1396.7926	-3.01	0	73	4.4e-05	1		K.AGLENGVLTVTVPK.A

Query	Start – End	Observed	Mr(expt)	Mr(calc)	ppm	M	Score	Expect	Rank	U	Peptide
715	123 – 136	699.4015	1396.7884	1396.7926	-2.99	0	72	5.8e-05	1		K.AGLENGVLTVTVPK.A
716	123 – 136	699.4017	1396.7889	1396.7926	-2.65	0	73	4.9e-05	1		K.AGLENGVLTVTVPK.A
717	123 – 136	699.4020	1396.7894	1396.7926	-2.29	0	64	0.00035	1		K.AGLENGVLTVTVPK.A
718	123 – 136	699.4021	1396.7895	1396.7926	-2.18	0	93	4.7e-07	1		K.AGLENGVLTVTVPK.A
719	123 – 136	699.4022	1396.7898	1396.7926	-1.98	0	89	1e-06	1		K.AGLENGVLTVTVPK.A
720	123 – 136	466.6040	1396.7901	1396.7926	-1.77	0	52	0.0053	1		K.AGLENGVLTVTVPK.A
721	123 – 136	699.4025	1396.7904	1396.7926	-1.53	0	66	0.00024	1		K.AGLENGVLTVTVPK.A

Spot no. 8

Ions score is -10*Log(P), where P is the probability that the observed match is a random event. Individual ions scores > 43 indicate identity or extensive homology (p<0.05). Protein scores are derived from ions scores as a non-probabilistic basis for ranking protein hits.

Alpha-amylase/trypsin inhibitor CM3 [*Triticum aestivum*]

Query	Start – End	Observed	Mr(expt)	Mr(calc)	ppm	M	Score	Expect	Rank	U	Peptide
1018	45 – 60	901.4259	1800.8373	1800.8353	1.13	0	111	9.4e-09	1		R.DYVLQQTCGTFTPGSK.L
1080	81 – 95	653.2898	1956.8477	1956.8492	-0.76	0	78	8.3e-06	1	U	K.LYCCQELAEISQQCR.C
1081	81 – 95	979.4319	1956.8492	1956.8492	0.013	0	100	6e-08	1	U	K.LYCCQELAEISQQCR.C
986	101 – 115	849.9636	1697.9126	1697.9141	-0.87	0	64	0.00051	1		R.YFIALPVPSQPVDPR.S
997	116 – 132	864.4204	1726.8262	1726.8308	-2.69	0	120	1.6e-09	1		R.SGNVGESGLIDLPGCPR.E
1047	141 – 157	938.5149	1875.0153	1875.0149	0.24	0	98	1.6e-07	1		R.LLVAPGQCNLATIHNVR.Y

Spot no. 9

Ions score is -10*Log(P), where P is the probability that the observed match is a random event. Individual ions scores > 43 indicate identity or extensive homology (p<0.05). Protein scores are derived from ions scores as a non-probabilistic basis for ranking protein hits.

0.19 Alpha-amylase inhibitor [*Triticum aestivum*]

Query	Start – End	Observed	Mr(expt)	Mr(calc)	ppm	M	Score	Expect	Rank	U	Peptide
837	26 – 39	785.9045	1569.7944	1569.7933	0.67	0	100	1.4e-07	1		R.LQCNGSQVPEAVLR.D
835	54 – 66	785.8337	1569.6528	1569.6513	0.97	0	59	0.00047	1		R.CGALYSMLDSMYK.E + 2 Oxidation (M)
862	67 – 82	806.8779	1611.7413	1611.7390	1.45	0	129	1.5e-10	1		K.EHGAQEGQAGTGAFPR.C

Query	Start	– End	Observed	Mr(expt)	Mr(calc)	ppm	M	Score	Expect	Rank	U	Peptide
460	90	– 100	581.8139	1161.6133	1161.6176	-3.66	0	85	6e-06	1		K.LTAASITAVCR.L
883	101	– 116	832.4227	1662.8309	1662.8287	1.32	0	119	2.2e-09	1		R.LPIVVDASGDGAYVCK.D

Spot no. 10

Ions score is -10*Log(P), where P is the probability that the observed match is a random event. Individual ions scores > 43 indicate identity or extensive homology (p<0.05). Protein scores are derived from ions scores as a non-probabilistic basis for ranking protein hits.

0.19 dimeric alpha-amylase inhibitor [*Triticum aestivum*]

Query	Start	– End	Observed	Mr(expt)	Mr(calc)	ppm	M	Score	Expect	Rank	U	Peptide
927	1	– 13	790.3432	1578.6719	1578.6636	5.28	0	87	9.9e-07	1		-.SGPWMCYPGYAFK.V + Oxidation (M)
686	14	– 25	653.8854	1305.7563	1305.7591	-2.11	0	42	0.034	1		K.VPALPGCRPVLK.L
917	26	– 39	785.9035	1569.7925	1569.7933	-0.49	0	100	1.4e-07	1		K.LQCNGSQVPEAVLR.E
918	26	– 39	785.9155	1569.8164	1569.7933	14.7	0	57	0.0027	1		K.LQCNGSQVPEAVLR.E
1117	40	– 53	927.8813	1853.7481	1853.7495	-0.73	0	98	3.6e-08	1	U	R.ECCQQLADISEWCR.C
852	54	– 66	769.8350	1537.6553	1537.6615	-3.99	0	80	5e-06	1		R.CGALYSMLDSMYK.E
863	54	– 66	777.8343	1553.6541	1553.6564	-1.50	0	88	7.6e-07	1		R.CGALYSMLDSMYK.E + Oxidation (M)
864	54	– 66	777.8349	1553.6553	1553.6564	-0.73	0	84	1.8e-06	1		R.CGALYSMLDSMYK.E + Oxidation (M)
915	54	– 66	785.8352	1569.6558	1569.6513	2.87	0	73	2e-05	1		R.CGALYSMLDSMYK.E + 2 Oxidation (M)
1138	67	– 84	629.9494	1886.8263	1886.8330	-3.56	0	53	0.0035	1		K.EHGVQEGQAGTGAFPSCR.R
1139	67	– 84	944.4213	1886.8280	1886.8330	-2.61	0	139	1e-11	1		K.EHGVQEGQAGTGAFPSCR.R
479	90	– 100	567.8120	1133.6094	1133.6114	-1.81	0	83	8.3e-06	1		K.LTAASITAVCK.L
1041	101	– 116	839.4254	1676.8363	1676.8444	-4.81	0	61	0.0013	1		K.LPIVIDASGDGAYVCK.D
1042	101	– 116	839.4269	1676.8392	1676.8444	-3.10	0	70	0.00017	1		K.LPIVIDASGDGAYVCK.D
1043	101	– 116	559.9544	1676.8414	1676.8444	-1.78	0	89	2.2e-06	1		K.LPIVIDASGDGAYVCK.D
1044	101	– 116	839.4283	1676.8421	1676.8444	-1.33	0	122	9.2e-10	1		K.LPIVIDASGDGAYVCK.D
1045	101	– 116	839.4285	1676.8424	1676.8444	-1.14	0	76	4.2e-05	1		K.LPIVIDASGDGAYVCK.D
1046	101	– 116	559.9555	1676.8445	1676.8444	0.097	0	67	0.00034	1		K.LPIVIDASGDGAYVCK.D
1047	101	– 116	839.4302	1676.8458	1676.8444	0.84	0	109	2.1e-08	1		K.LPIVIDASGDGAYVCK.D

Spot no. 11

Ions score is -10*Log(P), where P is the probability that the observed match is a random event. Individual ions scores > 43 indicate identity or extensive homology (p<0.05). Protein scores are derived from ions scores as a non-probabilistic basis for ranking protein hits.

monomeric alpha-amylase inhibitor [*Triticum aestivum*]

Query	Start – End	Observed	Mr(expt)	Mr(calc)	ppm	M	Score	Expect	Rank	U	Peptide
983	1 – 14	806.3451	1610.6757	1610.6824	-4.16	0	80	4.1e-06	1		-.SGPWSWCDPATGYK.V
984	1 – 14	806.3454	1610.6762	1610.6824	-3.84	0	71	3.8e-05	1		-.SGPWSWCDPATGYK.V
985	1 – 14	806.3457	1610.6768	1610.6824	-3.45	0	82	2.7e-06	1		-.SGPWSWCDPATGYK.V
986	1 – 14	806.3458	1610.6770	1610.6824	-3.37	0	75	1.2e-05	1		-.SGPWSWCDPATGYK.V
987	1 – 14	806.3459	1610.6772	1610.6824	-3.20	0	73	2.2e-05	1		-.SGPWSWCDPATGYK.V
988	1 – 14	806.3459	1610.6773	1610.6824	-3.18	0	75	1.3e-05	1		-.SGPWSWCDPATGYK.V
989	1 – 14	806.3464	1610.6782	1610.6824	-2.60	0	75	1.5e-05	1		-.SGPWSWCDPATGYK.V
990	1 – 14	806.3465	1610.6784	1610.6824	-2.48	0	73	2.4e-05	1		-.SGPWSWCDPATGYK.V
991	1 – 14	806.3469	1610.6792	1610.6824	-1.96	0	62	0.00028	1		-.SGPWSWCDPATGYK.V
992	1 – 14	806.3471	1610.6796	1610.6824	-1.71	0	75	1.5e-05	1		-.SGPWSWCDPATGYK.V
993	1 – 14	806.3471	1610.6797	1610.6824	-1.66	0	67	8.4e-05	1		-.SGPWSWCDPATGYK.V
994	1 – 14	806.3472	1610.6799	1610.6824	-1.56	0	75	1.4e-05	1		-.SGPWSWCDPATGYK.V
995	1 – 14	806.3473	1610.6801	1610.6824	-1.40	0	66	0.00011	1		-.SGPWSWCDPATGYK.V
996	1 – 14	1611.6877	1610.6804	1610.6824	-1.22	0	62	0.00028	1		-.SGPWSWCDPATGYK.V
997	1 – 14	806.3479	1610.6812	1610.6824	-0.73	0	77	1e-05	1		-.SGPWSWCDPATGYK.V
998	1 – 14	806.3481	1610.6816	1610.6824	-0.51	0	79	6.3e-06	1		-.SGPWSWCDPATGYK.V
999	1 – 14	806.3482	1610.6818	1610.6824	-0.35	0	91	4e-07	1		-.SGPWSWCDPATGYK.V
1000	1 – 14	806.3484	1610.6822	1610.6824	-0.087	0	70	5.4e-05	1		-.SGPWSWCDPATGYK.V
1001	1 – 14	806.3486	1610.6826	1610.6824	0.11	0	72	2.7e-05	1		-.SGPWSWCDPATGYK.V
1002	1 – 14	806.3494	1610.6843	1610.6824	1.20	0	75	1.7e-05	1		-.SGPWSWCDPATGYK.V
1003	1 – 14	806.3500	1610.6854	1610.6824	1.90	0	66	0.00012	1		-.SGPWSWCDPATGYK.V
1004	1 – 14	806.3501	1610.6855	1610.6824	1.96	0	76	1.4e-05	1		-.SGPWSWCDPATGYK.V
181	15 – 22	432.2224	862.4302	862.4331	-3.34	0	59	0.0031	1		K.VSALTGCR.A
184	15 – 22	432.2228	862.4311	862.4331	-2.34	0	48	0.033	1		K.VSALTGCR.A
902	27 – 40	778.4107	1554.8068	1554.8188	-7.75	0	73	7.8e-05	1		K.LQCVGSQVPEAVLR.D
903	27 – 40	778.4134	1554.8121	1554.8188	-4.29	0	84	5.2e-06	1		K.LQCVGSQVPEAVLR.D
904	27 – 40	778.4134	1554.8122	1554.8188	-4.24	0	93	6.5e-07	1		K.LQCVGSQVPEAVLR.D
905	27 – 40	778.4135	1554.8125	1554.8188	-4.08	0	87	3.1e-06	1		K.LQCVGSQVPEAVLR.D
906	27 – 40	778.4139	1554.8132	1554.8188	-3.62	0	79	1.7e-05	1		K.LQCVGSQVPEAVLR.D
907	27 – 40	778.4139	1554.8133	1554.8188	-3.53	0	87	2.6e-06	1		K.LQCVGSQVPEAVLR.D
908	27 – 40	778.4140	1554.8134	1554.8188	-3.47	0	77	2.9e-05	1		K.LQCVGSQVPEAVLR.D

Query	Start – End	Observed	Mr(expt)	Mr(calc)	ppm	M	Score	Expect	Rank	U	Peptide
909	27 – 40	778.4141	1554.8136	1554.8188	-3.35	0	75	4e-05	1		K.LQCVGSQVPEAVLR.D
910	27 – 40	778.4143	1554.8141	1554.8188	-3.04	0	92	8.9e-07	1		K.LQCVGSQVPEAVLR.D
911	27 – 40	778.4144	1554.8141	1554.8188	-3.00	0	95	4.5e-07	1		K.LQCVGSQVPEAVLR.D
912	27 – 40	778.4144	1554.8142	1554.8188	-2.96	0	89	1.6e-06	1		K.LQCVGSQVPEAVLR.D
914	27 – 40	778.4149	1554.8151	1554.8188	-2.36	0	76	3.4e-05	1		K.LQCVGSQVPEAVLR.D
915	27 – 40	778.4150	1554.8154	1554.8188	-2.21	0	70	0.00013	1		K.LQCVGSQVPEAVLR.D
916	27 – 40	778.4151	1554.8156	1554.8188	-2.06	0	78	2.3e-05	1		K.LQCVGSQVPEAVLR.D
917	27 – 40	778.4153	1554.8160	1554.8188	-1.81	0	84	5.7e-06	1		K.LQCVGSQVPEAVLR.D
918	27 – 40	778.4153	1554.8161	1554.8188	-1.72	0	80	1.5e-05	1		K.LQCVGSQVPEAVLR.D
919	27 – 40	778.4153	1554.8161	1554.8188	-1.72	0	81	1.1e-05	1		K.LQCVGSQVPEAVLR.D
920	27 – 40	778.4155	1554.8164	1554.8188	-1.55	0	92	9.8e-07	1		K.LQCVGSQVPEAVLR.D
921	27 – 40	778.4156	1554.8167	1554.8188	-1.38	0	79	1.6e-05	1		K.LQCVGSQVPEAVLR.D
922	27 – 40	778.4159	1554.8172	1554.8188	-1.06	0	92	8.8e-07	1		K.LQCVGSQVPEAVLR.D
923	27 – 40	1555.8247	1554.8174	1554.8188	-0.90	0	60	0.0015	1		K.LQCVGSQVPEAVLR.D
924	27 – 40	778.4160	1554.8175	1554.8188	-0.84	0	95	4.9e-07	1		K.LQCVGSQVPEAVLR.D
925	27 – 40	778.4167	1554.8188	1554.8188	-0.019	0	75	4.4e-05	1		K.LQCVGSQVPEAVLR.D
926	27 – 40	778.4168	1554.8190	1554.8188	0.11	0	100	1.3e-07	1		K.LQCVGSQVPEAVLR.D
927	27 – 40	778.4171	1554.8197	1554.8188	0.57	0	81	9.9e-06	1		K.LQCVGSQVPEAVLR.D
928	27 – 40	778.4178	1554.8211	1554.8188	1.46	0	76	3.2e-05	1		K.LQCVGSQVPEAVLR.D
929	27 – 40	778.4180	1554.8214	1554.8188	1.69	0	71	0.00012	1		K.LQCVGSQVPEAVLR.D
930	27 – 40	778.4188	1554.8230	1554.8188	2.70	0	79	1.8e-05	1		K.LQCVGSQVPEAVLR.D
931	27 – 40	778.4194	1554.8243	1554.8188	3.52	0	71	0.00011	1		K.LQCVGSQVPEAVLR.D
1223	41 – 55	991.3951	1980.7756	1980.7877	-6.06	0	85	3.4e-07	1		R.DCCQQLADINNEWCR.C
1224	41 – 55	991.3961	1980.7776	1980.7877	-5.07	0	90	1.3e-07	1		R.DCCQQLADINNEWCR.C
1225	41 – 55	991.3979	1980.7812	1980.7877	-3.25	0	93	7e-08	1		R.DCCQQLADINNEWCR.C
1226	41 – 55	991.3979	1980.7812	1980.7877	-3.24	0	108	2e-09	1		R.DCCQQLADINNEWCR.C
1227	41 – 55	991.3980	1980.7815	1980.7877	-3.10	0	104	5.1e-09	1		R.DCCQQLADINNEWCR.C
1228	41 – 55	991.3987	1980.7829	1980.7877	-2.40	0	98	2.1e-08	1		R.DCCQQLADINNEWCR.C
1229	41 – 55	991.3989	1980.7831	1980.7877	-2.28	0	108	2.3e-09	1		R.DCCQQLADINNEWCR.C
1230	41 – 55	661.2684	1980.7833	1980.7877	-2.22	0	76	3.1e-06	1		R.DCCQQLADINNEWCR.C
1231	41 – 55	991.3992	1980.7838	1980.7877	-1.95	0	90	1.4e-07	1		R.DCCQQLADINNEWCR.C
1232	41 – 55	991.3993	1980.7840	1980.7877	-1.83	0	95	4.4e-08	1		R.DCCQQLADINNEWCR.C
1233	41 – 55	991.3994	1980.7843	1980.7877	-1.70	0	107	2.5e-09	1		R.DCCQQLADINNEWCR.C

Query	Start	– End	Observed	Mr(expt)	Mr(calc)	ppm	M	Score	Expect	Rank	U	Peptide
1234	41	– 55	991.3998	1980.7850	1980.7877	-1.34	0	85	4.3e-07	1		R.DCCQQLADINNEWCR.C
1235	41	– 55	991.4002	1980.7859	1980.7877	-0.88	0	95	4.4e-08	1		R.DCCQQLADINNEWCR.C
1236	41	– 55	661.2693	1980.7860	1980.7877	-0.82	0	68	2.5e-05	1		R.DCCQQLADINNEWCR.C
1237	41	– 55	991.4003	1980.7861	1980.7877	-0.77	0	103	6.4e-09	1		R.DCCQQLADINNEWCR.C
1238	41	– 55	661.2694	1980.7862	1980.7877	-0.73	0	41	0.011	1		R.DCCQQLADINNEWCR.C
1239	41	– 55	661.2694	1980.7864	1980.7877	-0.63	0	36	0.038	1		R.DCCQQLADINNEWCR.C
1240	41	– 55	991.4010	1980.7875	1980.7877	-0.086	0	104	6.3e-09	1		R.DCCQQLADINNEWCR.C
1241	41	– 55	991.4011	1980.7876	1980.7877	-0.026	0	107	2.6e-09	1		R.DCCQQLADINNEWCR.C
1242	41	– 55	991.4013	1980.7881	1980.7877	0.21	0	96	3.9e-08	1		R.DCCQQLADINNEWCR.C
1243	41	– 55	991.4018	1980.7890	1980.7877	0.67	0	93	7.6e-08	1		R.DCCQQLADINNEWCR.C
1244	41	– 55	661.2705	1980.7897	1980.7877	1.03	0	48	0.0024	1		R.DCCQQLADINNEWCR.C
1245	41	– 55	991.4026	1980.7907	1980.7877	1.54	0	93	8.5e-08	1		R.DCCQQLADINNEWCR.C
1246	41	– 55	991.4027	1980.7908	1980.7877	1.61	0	108	2.2e-09	1		R.DCCQQLADINNEWCR.C
1247	41	– 55	661.2709	1980.7909	1980.7877	1.62	0	56	0.00036	1		R.DCCQQLADINNEWCR.C
1248	41	– 55	661.2709	1980.7910	1980.7877	1.68	0	42	0.0098	1		R.DCCQQLADINNEWCR.C
1249	41	– 55	991.4032	1980.7919	1980.7877	2.16	0	103	8e-09	1		R.DCCQQLADINNEWCR.C
1250	41	– 55	661.2716	1980.7928	1980.7877	2.61	0	55	0.00054	1		R.DCCQQLADINNEWCR.C
1252	41	– 55	991.4042	1980.7938	1980.7877	3.11	0	92	1e-07	1		R.DCCQQLADINNEWCR.C
1253	41	– 55	991.4043	1980.7940	1980.7877	3.19	0	103	7.8e-09	1		R.DCCQQLADINNEWCR.C
1254	41	– 55	991.4047	1980.7949	1980.7877	3.64	0	74	7e-06	1		R.DCCQQLADINNEWCR.C
1255	41	– 55	991.4048	1980.7951	1980.7877	3.74	0	85	5.4e-07	1		R.DCCQQLADINNEWCR.C
1256	41	– 55	991.4056	1980.7966	1980.7877	4.52	0	99	2.1e-08	1		R.DCCQQLADINNEWCR.C
384	56	– 64	519.7373	1037.4601	1037.4634	-3.17	0	66	0.00026	1		R.CGDLSSMLR.S
388	56	– 64	519.7384	1037.4623	1037.4634	-1.07	0	53	0.0051	1		R.CGDLSSMLR.S
437	56	– 64	527.7346	1053.4546	1053.4583	-3.54	0	60	0.00075	1		R.CGDLSSMLR.S + Oxidation (M)
440	56	– 64	527.7358	1053.4570	1053.4583	-1.26	0	45	0.028	1		R.CGDLSSMLR.S + Oxidation (M)
401	65	– 73	525.7801	1049.5457	1049.5506	-4.62	0	55	0.0071	1		R.SVYQELGVR.E
403	65	– 73	525.7807	1049.5469	1049.5506	-3.49	0	51	0.015	1		R.SVYQELGVR.E
404	65	– 73	525.7808	1049.5470	1049.5506	-3.41	0	58	0.0029	1		R.SVYQELGVR.E
405	65	– 73	525.7809	1049.5472	1049.5506	-3.21	0	60	0.0022	1		R.SVYQELGVR.E
406	65	– 73	525.7809	1049.5472	1049.5506	-3.15	0	59	0.0028	1		R.SVYQELGVR.E
407	65	– 73	525.7809	1049.5473	1049.5506	-3.09	0	68	0.00031	1		R.SVYQELGVR.E
409	65	– 73	525.7812	1049.5479	1049.5506	-2.54	0	66	0.00056	1		R.SVYQELGVR.E

Query	Start – End		Observed	Mr(expt)	Mr(calc)	ppm	M	Score	Expect	Rank	U	Peptide
410	65	– 73	525.7813	1049.5481	1049.5506	-2.31	0	66	0.00053	1		R.SVYQELGVR.E
411	65	– 73	525.7814	1049.5483	1049.5506	-2.12	0	59	0.0023	1		R.SVYQELGVR.E
412	65	– 73	525.7815	1049.5484	1049.5506	-2.08	0	52	0.013	1		R.SVYQELGVR.E
413	65	– 73	525.7816	1049.5487	1049.5506	-1.78	0	54	0.0074	1		R.SVYQELGVR.E
414	65	– 73	525.7817	1049.5488	1049.5506	-1.66	0	67	0.00038	1		R.SVYQELGVR.E
415	65	– 73	525.7817	1049.5488	1049.5506	-1.64	0	57	0.004	1		R.SVYQELGVR.E
416	65	– 73	525.7817	1049.5489	1049.5506	-1.57	0	57	0.0038	1		R.SVYQELGVR.E
417	65	– 73	525.7818	1049.5490	1049.5506	-1.51	0	55	0.0062	1		R.SVYQELGVR.E
418	65	– 73	525.7818	1049.5490	1049.5506	-1.45	0	73	0.00011	1		R.SVYQELGVR.E
419	65	– 73	525.7819	1049.5492	1049.5506	-1.30	0	53	0.0099	1		R.SVYQELGVR.E
420	65	– 73	525.7819	1049.5492	1049.5506	-1.24	0	59	0.0027	1		R.SVYQELGVR.E
421	65	– 73	525.7819	1049.5492	1049.5506	-1.24	0	65	0.00058	1		R.SVYQELGVR.E
422	65	– 73	525.7821	1049.5495	1049.5506	-0.96	0	62	0.0014	1		R.SVYQELGVR.E
423	65	– 73	525.7821	1049.5495	1049.5506	-0.96	0	62	0.0012	1		R.SVYQELGVR.E
424	65	– 73	525.7821	1049.5496	1049.5506	-0.94	0	51	0.018	1		R.SVYQELGVR.E
425	65	– 73	525.7821	1049.5497	1049.5506	-0.79	0	48	0.03	1		R.SVYQELGVR.E
426	65	– 73	525.7822	1049.5499	1049.5506	-0.59	0	59	0.0022	1		R.SVYQELGVR.E
428	65	– 73	525.7825	1049.5504	1049.5506	-0.18	0	57	0.0037	1		R.SVYQELGVR.E
429	65	– 73	525.7832	1049.5518	1049.5506	1.22	0	59	0.0025	1		R.SVYQELGVR.E
430	65	– 73	525.7832	1049.5519	1049.5506	1.31	0	62	0.0013	1		R.SVYQELGVR.E
431	65	– 73	525.7835	1049.5524	1049.5506	1.73	0	60	0.0022	1		R.SVYQELGVR.E
584	89	– 99	587.8077	1173.6009	1173.6064	-4.67	0	55	0.0057	1		K.LTAASVPEVCK.V
586	89	– 99	587.8083	1173.6021	1173.6064	-3.65	0	58	0.0029	1		K.LTAASVPEVCK.V
587	89	– 99	587.8085	1173.6024	1173.6064	-3.38	0	53	0.01	1		K.LTAASVPEVCK.V
588	89	– 99	587.8085	1173.6024	1173.6064	-3.35	0	76	4.9e-05	1		K.LTAASVPEVCK.V
589	89	– 99	587.8085	1173.6025	1173.6064	-3.26	0	69	0.00023	1		K.LTAASVPEVCK.V
590	89	– 99	587.8085	1173.6025	1173.6064	-3.24	0	60	0.0022	1		K.LTAASVPEVCK.V
592	89	– 99	587.8089	1173.6031	1173.6064	-2.73	0	54	0.0072	1		K.LTAASVPEVCK.V
593	89	– 99	587.8089	1173.6033	1173.6064	-2.56	0	59	0.0023	1		K.LTAASVPEVCK.V
594	89	– 99	587.8091	1173.6036	1173.6064	-2.37	0	59	0.0023	1		K.LTAASVPEVCK.V
595	89	– 99	587.8092	1173.6038	1173.6064	-2.17	0	57	0.0042	1		K.LTAASVPEVCK.V
596	89	– 99	587.8092	1173.6039	1173.6064	-2.12	0	50	0.019	1		K.LTAASVPEVCK.V
597	89	– 99	587.8092	1173.6039	1173.6064	-2.07	0	53	0.011	1		K.LTAASVPEVCK.V

Query	Start – End	Observed	Mr(expt)	Mr(calc)	ppm	M	Score	Expect	Rank	U	Peptide
598	89 – 99	587.8093	1173.6041	1173.6064	-1.90	0	52	0.012	1		K.LTAASVPEVCK.V
599	89 – 99	587.8095	1173.6044	1173.6064	-1.68	0	69	0.00024	1		K.LTAASVPEVCK.V
600	89 – 99	587.8095	1173.6045	1173.6064	-1.57	0	60	0.0022	1		K.LTAASVPEVCK.V
601	89 – 99	587.8095	1173.6045	1173.6064	-1.57	0	54	0.0089	1		K.LTAASVPEVCK.V
602	89 – 99	587.8096	1173.6046	1173.6064	-1.45	0	57	0.004	1		K.LTAASVPEVCK.V
604	89 – 99	587.8097	1173.6048	1173.6064	-1.35	0	50	0.019	1		K.LTAASVPEVCK.V
605	89 – 99	587.8097	1173.6048	1173.6064	-1.32	0	64	0.00083	1		K.LTAASVPEVCK.V
606	89 – 99	587.8097	1173.6048	1173.6064	-1.30	0	74	7.5e-05	1		K.LTAASVPEVCK.V
608	89 – 99	587.8098	1173.6049	1173.6064	-1.20	0	61	0.0016	1		K.LTAASVPEVCK.V
609	89 – 99	587.8098	1173.6051	1173.6064	-1.03	0	49	0.028	1		K.LTAASVPEVCK.V
610	89 – 99	587.8099	1173.6053	1173.6064	-0.89	0	62	0.0013	1		K.LTAASVPEVCK.V
612	89 – 99	587.8100	1173.6055	1173.6064	-0.70	0	52	0.014	1		K.LTAASVPEVCK.V
613	89 – 99	587.8103	1173.6060	1173.6064	-0.26	0	66	0.00055	1		K.LTAASVPEVCK.V
614	89 – 99	587.8106	1173.6065	1173.6064	0.17	0	57	0.0041	1		K.LTAASVPEVCK.V
615	89 – 99	587.8108	1173.6071	1173.6064	0.68	0	72	0.00014	1		K.LTAASVPEVCK.V
616	89 – 99	587.8112	1173.6078	1173.6064	1.27	0	55	0.007	1		K.LTAASVPEVCK.V
617	89 – 99	587.8115	1173.6085	1173.6064	1.85	0	59	0.0029	1		K.LTAASVPEVCK.V
618	89 – 99	587.8116	1173.6086	1173.6064	1.94	0	64	0.00079	1		K.LTAASVPEVCK.V
619	89 – 99	587.8118	1173.6091	1173.6064	2.35	0	67	0.0004	1		K.LTAASVPEVCK.V
1406	100 – 121	1153.0275	2304.0404	2304.0521	-5.07	0	53	0.0042	1		K.VPIPNPSGDGAGVCYWAAYPDV.-
1407	100 – 121	1153.0285	2304.0424	2304.0521	-4.21	0	103	3.9e-08	1		K.VPIPNPSGDGAGVCYWAAYPDV.-
1408	100 – 121	769.0216	2304.0429	2304.0521	-4.02	0	60	0.00084	1		K.VPIPNPSGDGAGVCYWAAYPDV.-
1409	100 – 121	1153.0292	2304.0438	2304.0521	-3.60	0	102	5.3e-08	1		K.VPIPNPSGDGAGVCYWAAYPDV.-
1410	100 – 121	1153.0299	2304.0452	2304.0521	-2.99	0	50	0.0087	1		K.VPIPNPSGDGAGVCYWAAYPDV.-
1411	100 – 121	769.0229	2304.0468	2304.0521	-2.31	0	59	0.001	1		K.VPIPNPSGDGAGVCYWAAYPDV.-
1412	100 – 121	1153.0308	2304.0470	2304.0521	-2.21	0	105	2.5e-08	1		K.VPIPNPSGDGAGVCYWAAYPDV.-
1413	100 – 121	769.0230	2304.0472	2304.0521	-2.13	0	51	0.0058	1		K.VPIPNPSGDGAGVCYWAAYPDV.-
1414	100 – 121	769.0231	2304.0476	2304.0521	-1.99	0	65	0.00024	1		K.VPIPNPSGDGAGVCYWAAYPDV.-
1415	100 – 121	1153.0311	2304.0476	2304.0521	-1.95	0	115	2.7e-09	1		K.VPIPNPSGDGAGVCYWAAYPDV.-
1416	100 – 121	1153.0314	2304.0482	2304.0521	-1.69	0	95	2.4e-07	1		K.VPIPNPSGDGAGVCYWAAYPDV.-
1417	100 – 121	1153.0316	2304.0486	2304.0521	-1.52	0	78	1.2e-05	1		K.VPIPNPSGDGAGVCYWAAYPDV.-
1418	100 – 121	769.0237	2304.0492	2304.0521	-1.30	0	82	4.8e-06	1		K.VPIPNPSGDGAGVCYWAAYPDV.-
1419	100 – 121	1153.0319	2304.0492	2304.0521	-1.25	0	107	1.7e-08	1		K.VPIPNPSGDGAGVCYWAAYPDV.-

Query	Start – End		Observed	Mr(expt)	Mr(calc)	ppm	M	Score	Expect	Rank	U	Peptide
1420	100	– 121	1153.0320	2304.0494	2304.0521	-1.17	0	76	1.9e-05	1		K.VPIPNPSGDGAGVCYWAAYPDV.-
1421	100	– 121	769.0238	2304.0497	2304.0521	-1.06	0	75	2.3e-05	1		K.VPIPNPSGDGAGVCYWAAYPDV.-
1422	100	– 121	1153.0329	2304.0512	2304.0521	-0.39	0	89	1.1e-06	1		K.VPIPNPSGDGAGVCYWAAYPDV.-
1423	100	– 121	1153.0330	2304.0514	2304.0521	-0.30	0	53	0.0043	1		K.VPIPNPSGDGAGVCYWAAYPDV.-
1425	100	– 121	1153.0332	2304.0518	2304.0521	-0.13	0	107	1.7e-08	1		K.VPIPNPSGDGAGVCYWAAYPDV.-
1426	100	– 121	769.0248	2304.0526	2304.0521	0.19	0	48	0.012	1		K.VPIPNPSGDGAGVCYWAAYPDV.-
1428	100	– 121	1153.0339	2304.0532	2304.0521	0.48	0	91	7e-07	1		K.VPIPNPSGDGAGVCYWAAYPDV.-
1429	100	– 121	1153.0339	2304.0532	2304.0521	0.48	0	66	0.00021	1		K.VPIPNPSGDGAGVCYWAAYPDV.-
1430	100	– 121	1153.0340	2304.0534	2304.0521	0.57	0	115	2.9e-09	1		K.VPIPNPSGDGAGVCYWAAYPDV.-
1431	100	– 121	769.0258	2304.0556	2304.0521	1.52	0	52	0.0057	1		K.VPIPNPSGDGAGVCYWAAYPDV.-
1434	100	– 121	769.0267	2304.0583	2304.0521	2.69	0	54	0.0039	1		K.VPIPNPSGDGAGVCYWAAYPDV.-
1435	100	– 121	769.0274	2304.0603	2304.0521	3.52	0	61	0.00074	1		K.VPIPNPSGDGAGVCYWAAYPDV.-
