# Supplementary material for: Identification of Kernel Proteins Associated with the Resistance to Fusarium Head Blight in Winter Wheat (Triticum aestivum L.)
Source: PLoS One. 2014 Oct 23;9(10):e110822. doi: 10.1371/journal.pone.0110822 (PMC4207761; doi:10.1371/journal.pone.0110822)
Supplement: Table S1 — Meteorological conditions (sum of rainfalls and mean temperature) during the experiments performed in Cerekwica and Radzikow in 2013. (PDF) [file pone.0110822.s006.pdf]

**Table S1.** Meteorological conditions (sum of rainfalls and mean temperature) during the experiments performed in Cerekwica and Radzikow in 2013.

| Location | Rainfall [mm] | | Mean temperature [°C] | |
|---|---|---|---|---|
| | June | July | June | July |
| Cerekwica | 121.4 | 45.0 | 18.1 | 20.7 |
| Radzikow | 113.8 | 23.4 | 18.7 | 20.0 |
